# Functional Network Overlap as Revealed by fMRI Using sICA and Its Potential Relationships with Functional Heterogeneity, Balanced Excitation and Inhibition, and Sparseness of Neuron Activity

**DOI:** 10.1371/journal.pone.0117029

**Published:** 2015-02-25

**Authors:** Jiansong Xu, Vince D. Calhoun, Patrick D. Worhunsky, Hui Xiang, Jian Li, John T. Wall, Godfrey D. Pearlson, Marc N. Potenza

**Affiliations:** 1 Department of Psychiatry, Yale University School of Medicine, New Haven, Connecticut, United States of America; 2 Child Study Center, Yale University School of Medicine, New Haven, Connecticut, United States of America; 3 Department of Neurobiology, Yale University School of Medicine, New Haven, Connecticut, United States of America; 4 The Mind Research Network, Albuquerque, New Mexico, United States of America; 5 Olin Neuropsychiatry Research Center, Institute of Living, Hartford, Connecticut, United States of America; 6 Dept of ECE, The University of New Mexico, Albuquerque, New Mexico, United States of America; 7 Dept of Psychiatry, Guizhou Provincial People’s Hospital, Guiyang, Guizhou Province, P. R. China; 8 Dept of Neurosciences, University of Toledo, Toledo, Ohio, United States of America; 9 Department of Radiology, The First Hospital of China Medical University, Shenyang, Liaoning, P. R. China; National Scientific and Technical Research Council (CONICET)., ARGENTINA

## Abstract

Functional magnetic resonance imaging (fMRI) studies traditionally use general linear model-based analysis (GLM-BA) and regularly report task-related activation, deactivation, or no change in activation in separate brain regions. However, several recent fMRI studies using spatial independent component analysis (sICA) find extensive overlap of functional networks (FNs), each exhibiting different task-related modulation (e.g., activation vs. deactivation), different from the dominant findings of GLM-BA. This study used sICA to assess overlap of FNs extracted from four datasets, each related to a different cognitive task. FNs extracted from each dataset overlapped with each other extensively across most or all brain regions and showed task-related concurrent increases, decreases, or no changes in activity. These findings indicate that neural substrates showing task-related concurrent but different modulations in activity intermix with each other and distribute across most of the brain. Furthermore, spatial correlation analyses found that most FNs were highly consistent in spatial patterns across different datasets. This finding indicates that these FNs probably reflect large-scale patterns of task-related brain activity. We hypothesize that FN overlaps as revealed by sICA might relate to functional heterogeneity, balanced excitation and inhibition, and population sparseness of neuron activity, three fundamental properties of the brain. These possibilities deserve further investigation.

## Introduction

Blood oxygenation-level dependent (BOLD) functional magnetic resonance imaging (fMRI) studies traditionally use a general-linear-model-based analysis (GLM-BA) to interrogate BOLD time series. They often find task-related activation, deactivation, and no change in activation, with a mutually exclusive state associated with specific voxels within each brain region. While these findings help understand brain functional organization, they are not always consistent with data generated from other methods. For example, electrophysiological recordings from monkey brain often find that neurons showing task-related activation, deactivation, or no changes in activation intermix with each other in the same brain regions [1–4], in contrast to the separated activation and deactivation reported by fMRI using a GLM-BA. Several factors including limited spatial and temporal resolutions of fMRI and the univariate nature of GLM-BA may contribute to these different findings.

### Spatial independent component analysis & functional network overlap

Different from GLM-BA, spatial independent component analysis (sICA) is a multivariate approach and assumes that fMRI signal from each voxel represents a linear mixture of source signals; separates this signal mixture into spatially independent source signals using higher-order statistics; and groups all brain regions showing synchronized source signals into independent components (ICs) [5–7]. Therefore, all brain regions associated with an IC can be treated as an intrinsically coherent functional network (FN) with a unique timecourse. Relative to GLM-BA, a novel finding of sICA is extensive overlap of multiple FNs, even those showing task-related concurrent but opposite modulations (e.g., activation vs. deactivation) [8–10]. This finding is detected due to the strength of sICA in separating a signal mixture from each voxel into multiple source signals.

In one of its earliest applications in fMRI, sICA allowed separation of BOLD signal from one voxel into as many as six ICs [6]. Since then, multiple studies described spatial overlap of two or more FNs, indicating that sICA splits the BOLD signal from each voxel within overlapping regions into two or more FNs [11–19]. More recently, four different groups (including ours) have specifically assessed FN overlap using sICA implemented in the Group ICA Toolbox (GIFT) [5,20] or MELODIC [8–10,21–25]. Four of these studies from three different groups explicitly described task-related, concurrent, opposite modulations of overlapping FNs [8–10,26].

In the two studies from our group [10,26], GIFT was used to extract FNs from fMRI data related to visual target-identifying tasks with parametric task loads. In both studies, a group of FNs showed load-dependent increases in activity (e.g., FNs related to top-down attention control). These FNs overlapped with each other extensively and together covered the frontoparietal cortex, striatum, thalamus, and other cortical and subcortical regions. Another group of FNs showed load-dependent decreases in activity (e.g., FNs related to bottom-up stimulus induced reorientation). These FNs also overlapped with each other extensively and covered most cortical and subcortical regions. Even though the FNs in the two groups showed task-related, concurrent, and opposite modulations, they overlapped at both the lateral and medial frontoparietal cortex, anterior cingulate (ACC), insula, and temporal and occipital cortices.

A third study used GIFT to extract FNs from an fMRI dataset related to an anti-saccadic task and found extensive overlap of FNs showing task-related concurrent but different modulations [8]. This study specifically developed a tool for measuring the changes in BOLD signal of each FN within overlapping regions and confirmed the prediction that the sum of these measures from all overlapping FNs equaled to the measure of task-related changes in BOLD signal within the same regions as revealed by a GLM-BA. This study further reported that four FNs overlapped at the precuneus region. Two of them showed task-related activation while the remaining two FNs showed concurrent deactivation. Their activation and deactivation cancelled each other in the overlapping region so that a GLM-BA did not detect significant task-related changes in activity.

The most recent study used MELODIC to extract FNs from fMRI datasets investigating speech (Speech), counting (Count), and decision-making (Decision) [9]. They found that one FN at the left frontal-temporal-parietal (FTP) cortices increased activity during Speech but reduced activity during Count and Decision, and that another FN, mirroring the first FN at the right FTP, reduced activity during Speech but increased activity during Count and Decision. The two FNs overlapped with each other at the left parietal and frontal cortices even though they showed task-related, concurrent, opposite modulations. Furthermore, a third FN extensively overlapped with the FN at the left FTP and showed reduced activation during Speech and Count. Therefore, three overlapping FNs showed task-related, concurrent, opposite modulations during Speech and Count.

In summary, the main findings of the above reviewed studies include: 1) some FNs extracted by sICA overlap with each other extensively, 2) each of the overlapping FNs shows a unique timecourse and/or task-related modulation, 3) some overlapping FNs show opposite task-related modulation (i.e., activation vs. deactivation) simultaneously, and 4) GLM-BA may not detect task-related activity in these overlapping regions. These findings initially appear to be in conflict with the dominant existing fMRI data of separated activation and deactivation as revealed by GLM-BA, but they do provide an intuitive explanation for the observed brain activity. In addition, these findings of sICA are consistent with the intermixed activation and deactivation of different neurons in the same brain regions as revealed by electrophysiological recordings [1–4]. Furthermore, sICA has been applied to fMRI for more than 15 years and more than four thousand fMRI studies using sICA have been published [27]. These studies found at least 10 ∼ 15 ICs which are highly consistent in spatial pattern across different studies using different populations at various task conditions [14,28], indicating that these ICs probably reflect true large-scale patterns of brain activity. Therefore, the findings of FN overlap should not be dismissed as artifacts simply because they are different from the dominant GLM-BA findings and raise the question as to which brain and behavioral processes relate to these overlaps in ICs.

### Balanced excitation and inhibition & functional heterogeneity

Based on the afore-mentioned findings and the following discussions, we hypothesized that at least two functional properties of the brain as revealed by electrophysiological recordings and other methods might relate to FN overlap as revealed by sICA. The first is balanced excitation and inhibition (E/I). Within any cortical region, about 20% of all neurons are GABAergic inhibitory interneurons and 80% are excitatory pyramid neurons [29]. These inhibitory and excitatory neurons form dense connections and maintain a balance of E/I at levels of single neurons and microcircuits [30–35]. Spontaneous or stimulus-induced activation of cortical neurons is accompanied by deactivation of adjacent neurons and vice versa [36–39].

The second property is functional heterogeneity at cortical regions. Neurons within any cortical region including the primary sensory cortex are considerably heterogeneous in their functional properties [24,39–43]. For example, the primary visual cortex contains several overlapping regions or maps, each responsive to a unique visual property such as edge orientation, motion direction, and spatial frequency. These overlapping maps form a so-called ‘polymap’ [44–47]. The primary auditory and olfactory cortices also show “salt and pepper” like organization [39–41,48,49]. In the higher associative cortex such the prefrontal cortex (PFC), neurons responsive to any specific stimulus property constitute a minority at any region, with a relative higher density at some locations [50–52]. Therefore, any PFC region contains intermixed neurons with different functional properties. For example, during the delay period of a working memory task, different neurons intermixed in the same PFC regions may show different changes in activity including sustained increases, gradual increases, and sustained decreases, reflecting different cognitive processes such as cue representation, memory maintenance, and response preparation [1,3,52,53]. These data indicate that different FNs overlap with each other in the PFC [52].

A typical voxel in the cortex with 3∼5 mm in each dimension contains hundreds of thousands of neurons [29,54]. The above-discussed properties suggest that within each voxel at any instant, different neurons may show different functional activities (i.e., functional heterogeneity), and some neurons may increase activity while others may decrease activity simultaneously (i.e., balanced E/I). At the level of large-scale FNs, these properties suggest that: 1) multiple FNs may overlap with each other at any cortical region, with each possibly showing a unique timecourse (i.e., functional heterogeneity); and, 2) some FNs may show concurrent but opposite changes in activity (i.e., balanced E/I). These predicted features of brain functional organization are consistent with the FN overlap as revealed by sICA. Therefore, FN overlap as revealed by sICA may be interpreted as reflecting balanced E/I and functional heterogeneity in the brain and warrants further study.

### Aim of this study

For further understanding FN overlap and how it might relate to brain functional organization, this study assessed the overlap of FNs extracted from four fMRI datasets, each related to a different cognitive task. The extent of overlap in FNs exhibiting significant task-related activation or deactivation was determined, as was whether there existed no identifiable task-related changes in activation. This study also compared data revealed by sICA and a GLM-BA for comparing findings generated by the two different approaches. It also assessed “goodness of fit” of FNs extracted from different datasets for estimating their consistency in spatial patterns. Studying in this manner multiple datasets employing different tasks should provide insight into whether FN overlap reflects a general property of brain functional organization and/or whether these features of brain activity relate to specific tasks. We selected four specific tasks because they taxed different aspects of brain function and were expected to activate and deactivate different FNs.

## Materials and Methods

### Participants

**Flanker task**. This dataset was downloaded from https://openfmri.org (ds000102), a free open source for fMRI data [55–58]. Participants include 26 right-handed adults with a mean age of 28.1 years (standard deviation (SD) = 8.5).

**False belief task (FBT)**. This dataset was downloaded from https://openfmri.org (ds000109) [59]. It has two groups of healthy participants. The younger group consists of 21 subjects (mean age(years) = 22.7, SD = 0.9; range18–37; female = 15) while the older group consists of 12 subjects (mean age = 72 years; SD = 1.9; range: 65 ∼ 88; female = 5). The current study includes all subjects together as one group.

**Attention task**. This dataset has been described before [10]. It includes 28 healthy participants (ages 23–41 years, all right handed, 13 females). The original study for acquiring this dataset was approved by Institutional Review Board at University of California Los Angeles (UCLA). All the participants gave written informed consent.

**Monetary incentive delay task (MIDT)**. This dataset includes 70 healthy participants (mean age = 32.4 years, SD = 8.2; female = 31). The original study for acquiring this dataset was approved by Human Investigation Committee at Yale School of Medicine. All the participants gave written informed consent.

### Task Stimuli and Design

**Flanker task**. This is a slow-paced task with inter-trial intervals ranging from 8s to 14s (mean = 12s). During each trial, five arrows were presented at the screen center and participants were required to press one of two buttons to indicate the direction of the central arrow. All arrows pointed in the same direction in the congruent condition, and the central arrow pointed to the direction opposite to the direction of other four arrows in the incongruent condition. Therefore, the congruent condition is a simple display-response task, while the incongruent condition involves interference control (i.e., selective attention) and/or response inhibition.

**FBT**. Participants read short scripts and answered questions during this task. It requires the participants to remember the content and answer questions about the mental status of a character described in the script [59]. The task uses a block design with the duration of reading block being 10s, the duration of answering the question being 6s and a variable interval of 0–6s separating blocks of reading and answering questions. Each participant performed two functional runs, and each run included 6 scripts.

**Attention task**. The task uses a block design. Each block uses the same 16 faces, each of which was paired with a scene picture, as stimuli. The duration of each block was 19.2s (16 x 1.2s). The task has four levels of cognitive loads, one for rest (L0) and the remaining three for low (L1), medium (L2), and high (L3) levels of attention and working memory load. Participants rested during L0 and identified targets with 2, 3, and 4 facial features in conjunction during L1, L2, and L3, respectively [10]. Therefore, this task involves both sustained and selective attention.

**MIDT**. In this version of the MIDT [60], each trial of the task consists of two anticipatory phases (A1 and A2) and an outcome phase. During A1, participants anticipated to perform a task (pressing a button when a box appeared on-screen) for receiving a reward or avoiding a punishment. During A2, participants anticipated to receive a reward or avoid a punishment after performing the task (pressing a button). At the onset of each trial, a cue indicating win or loss of $0, $1, or $5 appears for 1s on the screen, with the onset of A1 occurring after cue presentation. Participants were required to press a button as soon as possible when a target appeared after a variable delay (1 to 3s) following the cue. If the participants pressed the button before the disappearance of the target, they won or did not lose the amount of money indicated by the cue; otherwise, they lost or did not win. Every participant performed two functional runs of the task, and each run consisted of 11 trials for each trial type.

### Imaging Data Acquisition

**Flanker task**. The fMRI data were acquired using a 3T scanner with TR = 2000 ms, TE = 30 ms, flip angle = 80°, and a voxel size of 3x3x4 mm^3^. Each participant performed two functional runs, and each run acquired 146 volumes.

**FBT**. Functional data were acquired using a 3T scanner with TR = 2000 ms, TE = 35ms, voxel size of 3x3x3 mm^3^, 0.54 mm skip, and 36 slice. Each participant had two runs and each run acquired 179 volumes.

**Attention task**. Functional images were acquired using gradient-echo EPI scanning sequence with a Siemens Allegra 3T system. TR = 1500 ms, TE = 30 ms, Flip angle = 70°, voxel size of 3x3.125x3.125 mm^3^, 1.2mm skip, 26 slices. Each participant had three functional runs, and each run acquired 258 volumes.

**MIDT**. Functional data were acquired using a 3T scanner and EPI gradient-echo sequence. TR = 1500 ms, TE = 27 ms, flip angle = 60°, voxel size of 3.4x3.4x4 mm^3^, 1mm skip, 25 slices. Each participant had two functional runs, and each run acquired 480 volumes.

### ICA procedures

Each BOLD time series was motion-corrected, normalized to the MNI (Montreal Neurological Institute) template, and smoothed with an 8-mm kernel using SPM5 (Statistical Parametric Mapping, Welcome Department of Cognitive Neurology, London). Group ICA algorithm (GIFT, http://icatb.sourceforge.net/, version1.3h) was used to extract spatially independent components (ICs) from each dataset separately [20,61]. Therefore, a total of four executions were performed. Data from all participants of each dataset were concatenated into a single dataset and reduced using two stages of principal component analysis (PCA) [61]. We extracted 75 ICs from each dataset by using Infomax algorithm [62]. The reason for using this high model order ICA is that it generates refined components consistent with known anatomical and functional segmentations of the brain [63–69]. Infomax algorithm generated a spatial map and a timecourse of the source signal changes for each IC. For each dataset, this analysis was repeated 50 times using ICASSO for assessing the repeatability of ICs [70] (Fig. A in S1 File). Finally, IC timecourses and spatial maps were back-reconstructed for each participant [61,71].

Two experienced investigators (Xu and Worhunsky) visually inspected each IC to separate artifacts from functional networks (FNs). Our diagnostic criteria for artifacts were consistent with the criteria used previously [63]. ICs with peak voxels in white matter and/or cerebrospinal fluid (CSF) were diagnosed as artifacts. Small dynamic range of power spectra and/or small ratio of the integral of spectral power below 0.10 Hz to the integral of power between 0.15 and 0.25 Hz were used as indicators of artifacts [63,72]. For defining brain regions associated with each IC, we used the GIFT one-sample t-test tool to create a group-level t-map for each IC. The significance threshold was set at voxel height p<.001, False-Discovery-Rate (FDR)-corrected for multiple comparisons of voxel-wise whole-brain analysis. Fig. B in S1 File shows spatial maps of most ICs diagnosed as functional networks.

### Assessing FN overlap

Since each IC has a positive and a negative element, the above-described t-map of each IC contains significant clusters with positive and negative t values. In this paper, we call the positive and negative clusters in the t-map as positive and negative sub-networks, respectively, and each sub-network represents one FN. In the following text of this paper, the term IC will be used to refer to a component extracted by sICA, and each IC includes both positive and negative sub-networks. In contrast, FN will be used to refer to either the positive or negative sub-network of each IC.

We converted each sub-network into a binary mask. Only significant voxels in each sub-network surviving the statistical threshold described above were converted into voxels with a value of one in the output mask, while all other voxels were converted into voxels with values of zero. Two masks were generated for each t-map: one for positive clusters and one for negative clusters. We added these masks together for assessing FN overlap in each dataset. Within the output map, any voxels with a value of two or higher number indicate that two or more sub-networks (i.e., FNs) overlap at this voxel.

### Assessing task-related modulation over timecourses

To examine task engagement of each IC, a design matrix for each participant was constructed using SPM5. This design matrix represents the onset of each trial or task block (details are presented in the next section). The temporal sorting function from GIFT was used to perform a multiple regression analysis between IC timecourse and the design matrix for each participant. For each IC, this regression generated a beta-weight value for each trial type of each functional run. These beta-weight values represent the correlations between IC timecourses and the canonical hemodynamic response model of task conditions, and index the engagement of ICs during specific task conditions [71]. An increase or decrease in beta-weight values in one task condition relative to another indicates an increase or decrease in task-related activity in the IC.

The beta-weights of each IC for each task condition across multiple runs of each subject were averaged, and then the group mean of averaged beta-weights for each task condition was tested against zero using SPSS one-sample t-tests. The significance threshold was p<.05, using FDR algorithm for correction of multiple comparisons due to multiple ICs. A positive beta-weight significantly different from zero indicates a task-related up-modulation of timecourse or increase in activity of the positive sub-network of the IC during a specific task condition relative to the baseline condition. Therefore, the positive sub-network of this IC was designated as a positive FN, and the negative sub-network as a negative FN. A significant negative beta-weight indicates a task-related down-modulation of timecourse or decrease in activity of the positive sub-network of the IC. Therefore, the positive sub-network of this IC was designated as a negative FN, and the negative sub-network as a positive FN. If the beta-weight was not significantly different from 0 after correction for multiple comparisons, then the IC is a neutral IC and both of its sub-networks are neutral FNs. Paired t-tests were used to compare beta-weights between two conditions within a task. The significance threshold was set at p<.05, using FDR algorithm for correction of multiple comparisons due to multiple ICs. Table A in S1 File shows the beta-weight and p values of each IC from each dataset. For assessing the volume of all positive FNs, we grouped all positive FNs into one brain space, and the total number of voxels covered by any positive FN represented the total volume of all positive FNs. A similar method was used to calculate the total volume of negative FNs and neutral FNs.

In the current study, the beta-weights in congruent conditions in the Flanker task were used to assess congruent condition-related FN modulation. The beta-weights related to reading false-belief scripts in the FBT were used to assess reading false-belief script-related FN modulation. The beta-weights in low- and high-load conditions in the attention task were used to assess low- and high-load condition-related FN modulation. The beta-weights in W1 and W5 conditions in the MIDT were used to assess W1- and W5-related FN modulation.

### Assessing “goodness of fit” of ICs extracted from different datasets

Spatial correlation tool provided with GIFT was used to assess “goodness of fit” of ICs extracted from the four datasets. This analysis will allow us to match ICs with similar spatial patterns from different datasets. Based on a recent publication [9], any pair of ICs was defined as highly correlated if their correlation coefficient (r) ≥ 0.5. Fig. B in S1 File shows all matched ICs with r ≥ 0.4.

### Assessing task-related changes in activity using SPM5

For GLM-based analyses, we used SPM5 to assess task-related changes in brain activity for each dataset. We used the same SPM5 design matrix created for temporal sorting as described above for these analyses. For the Flanker task, congruent and incongruent conditions were modeled as different event-related trials. For the FBT, scripts related to false belief and physical representations were modeled as different blocks, as were the blocks for answering related questions. For the attention task, the resting and low-, medium-, and high-load conditions were modeled as four different block conditions, while the instruction before each block was not explicitly modeled. For the MIDT, the trials of Win $0 and Loss $0 were used as baseline and not explicitly modeled. The first anticipation phase (A1) of trials for Win $1, Win $5, Loss $1, and Loss $5 were modeled as short blocks separately. The block durations were the intervals between cue onsets and target onsets. The second anticipation phase (A2) of trials for Win $1, Win $5, Loss $1, and Loss $5 were also modeled as short blocks separately. The block durations were the intervals between responses of participants and outcome onsets. The outcome phase of trials for Win $1, Win $5, Loss $1, and Loss $5 were modeled as event-related trials separately.

For the first-level analysis of each subject, a contrast map was created for each task condition to assess significant changes in BOLD signal between task conditions for each participant. The contrast images generated from each participant were inputted into a second-level (random-effect) one-sample t-test for group means. We employed cluster p<0.05, family-wise-error (FWE) corrected in conjunction with voxel-height threshold p<0.01 to identify task-related significant changes in BOLD signal.

## Results

In this paper, the voxels or FNs showing significant increases or decreases or no significant changes in BOLD signal during each task condition were termed as positive, negative, and neutral voxels or FNs, respectively. Below we first describe findings unique to each task and then findings across tasks.

### Findings unique to each task

**Fig 1:**
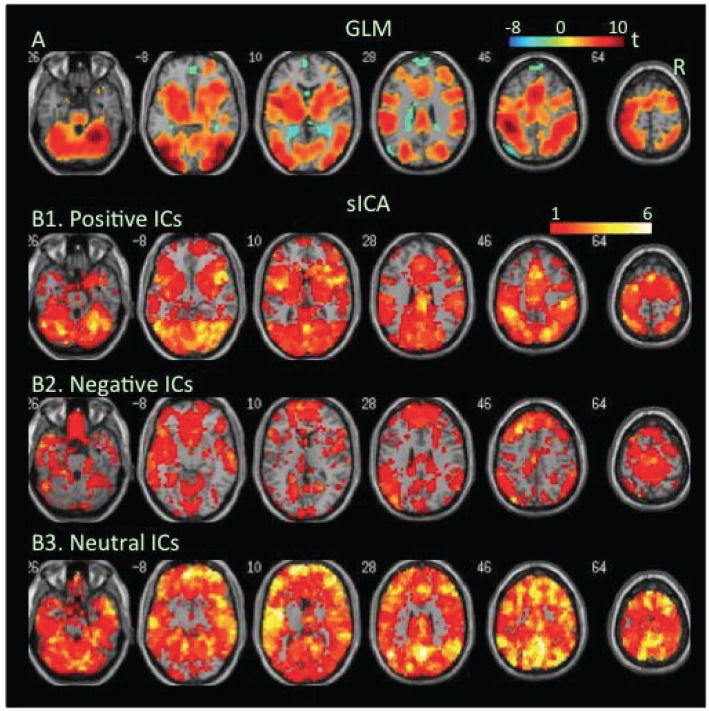
Task-related modulation in BOLD signal during the congruent condition of the flanker task. A. Color on the brain images shows task-related increases and decreases in BOLD signal as revealed by GLM-based analyses. The color bar indicates t values. B1, B2, and B3. Color on the brain images shows regions covered by positive, negative, and neutral ICs, respectively. The color bar indicates number of overlapping ICs.

**Flanker task**. During the congruent condition, extensive brain regions showed significant increases, but only a small cluster showed decreases in BOLD signal as revealed by the GLM-BA (Fig. 1A, Table 1, Table B in S1 File). However, sICA found extensive cortical and subcortical regions covered by negative FNs as well as positive FNs (Fig. 1B1–3, Tables 2 & 3).

**Table 1:** Brain volumes showing changes in BOLD signal.

	Posi Voxels	Posi Voxels Overlap With		NegaVoxels	Nega Voxels Overlap With		Overlap Posi & Nega Voxels and ICs
		Posi ICs	Nega ICs		NegaICs	PosiICs	
Flanker	31873 (52.7)	25113 (41.6)	9451 (15.6)	493 (0.8)	411(0.6)	88 (0.1)	26870 (44.5)
FBT	19464 (30.8)	15430 (24.4)	11154 (17.6)	5392 (8.5)	4936 (7.8)	3472 (5.5)	21577 (34.1)
Attention							
L1	0	0	0	5925 (9.5)	3679 (5.9)	1915 (3.1)	4242 (6.8)
L3	2268 (3.6)	2256 (3.6)	1675 (2.7)	12609 (20.3)	11970 (19.2)	8732 (14.0)	14382 (23.1)
L3—L1	4183 (6.7)	4138 (6.7)	2448 (3.9)	11841 (19.0)	11270 (18.1)	8185 (13.2)	15595 (25.1)
MIDT							
W1	8678 (13.4)	8599 (13.3)	8230 (12.7)	12081 (18.7)	11839 (18.3)	11517 (17.8)	20460 (31.6)
W5	16289 (25.1)	16068 (24.8)	15758 (24.3)	6018 (9.3)	5859 (9.0)	5686 (8.8)	22040 (34.0)
W5—W1	11318 (17.5)	9754 (15.1)	9159 (14.1)	0	0	0	10950 (16.9)

The unit of volume is voxel. Positive and negative indicate voxels or independent components (ICs) showing task-related increase and decreases, respectively, in BOLD signal. The number in parenthesis indicates % of the whole brain. Abbreviation: FBT: false belief task; nega: negative; posi: positive.

**Table 2:** Maximum overlap of different FNs of each dataset.

	All	Positive	Negative	Neutral
Flanker	13	6	5	9
FBT	17	9	9	8
Attention	15			
L1		5	6	12
L1		10	9	10
L3 vs. L1		9	8	8
MIDT	32			
W1		12	12	15
W5		13	13	12
W5 vs. W1		10	10	16

All, Positive, Negative, and Neutral columns list overlap of maximum numbers of all, positive, negative, and neutral FNs, respectively, of each dataset. FBT: false belief task.

**Table 3:** Brain volumes covered by different ICs.

	Total	Posi	Nega	Neut	PNTot	PN	PNN
Flanker	60401	38504 (63.7)	26464 (43.8)	52316 (86.6)	49970 (82.7)	14998 (24.8)	13281 (22.0)
FBT	63212	46861 (74.1)	48189 (76.2)	53406 (84.5)	57712 (91.3)	37338 (59.1)	30951 (49.0)
Attention	62183						
L1		18576 (29.9)	34510 (55.5)	61171 (98.4)	41235 (66.3)	11851 (19.1)	11672 (18.8)
L3		47852 (77.0)	51001 (82.0)	55442 (89.2)	59524 (95.2)	39329 (63.2)	38726 (62.3)
L3—L1		48746 (78.4)	48831 (78.5)	50310 (80.9)	59210 (95.2)	38367 (61.7)	31566 (50.8)
MIDT	64763						
W1		63056 (97.4)	62538 (96.6)	63524 (98.1)	64523 (99.6)	61080 (94.3)	60259 (93.0)
W5		62958 (97.2)	62979 (97.2)	63635 (98.3)	64410 (99.5)	61572 (95.1)	60672 (93.7)
W5—W1		61779 (95.4)	61524 (94.5)	64325 (99.3)	64410 (99.4)	61080 (94.3)	60259 (93.0)

The unit of volume is voxel. Total column lists total brain volume of each dataset. Posi, Nega, and Neut columns list numbers of voxels covered by positive ICs, negative ICs, and neutral ICs, respectively. PNTot, PN, and PNN columns list numbers of voxels covered by either positive or negative ICs, both positive-negative ICs, and all positive-negative-neutral ICs, respectively. The numbers in parentheses indicate % of whole-brain volumes. FBT: false belief task.

**Fig 2:**
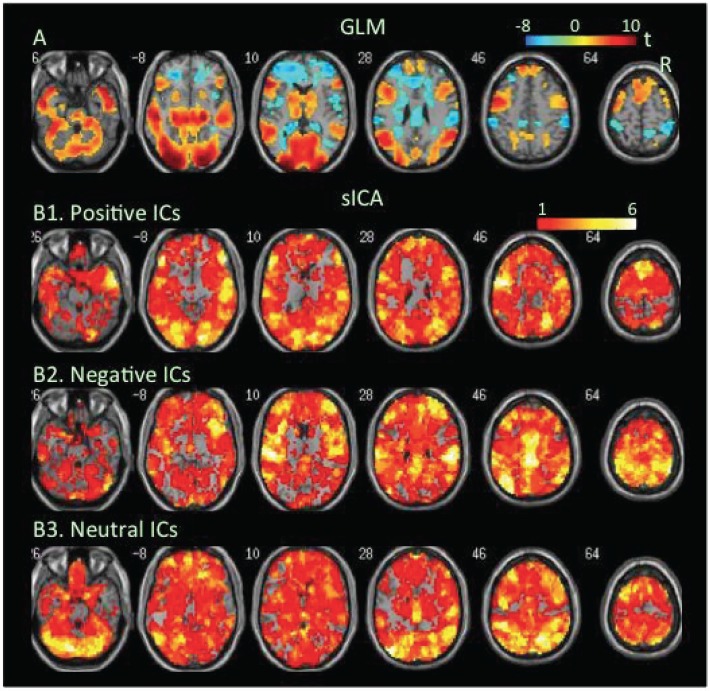
Task-related modulation in BOLD signal during reading false belief scripts. A. Color on the brain images shows task-related increases and decreases in BOLD signal as revealed by GLM-based analyses. The color bar indicates t values. B1, B2, and B3. Color on the brain images shows regions covered by positive, negative, and neutral ICs, respectively. The color bar indicates number of overlapping ICs.

**FBT**. The GLM-BA revealed task-related increases and decrease in BOLD signal in the medial and lateral frontoparietal cortex (FPC) during reading the false belief script (Fig. 2A, Table 1, Table B in S1 File), while sICA found more extensive cortical and subcortical regions covered by positive and negative FNs (Fig. 2B1–3, Tables 2 & 3).

**Fig 3:**
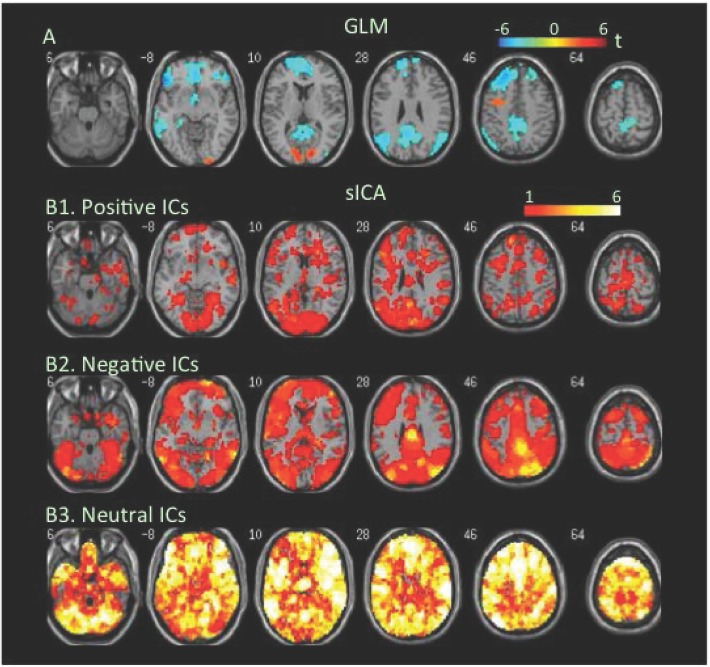
Task-related modulation in BOLD signal during the low-load condition relative to the baseline of the attention task. A. Color on the brain images shows task-related increases and decreases in BOLD signal as revealed by GLM-based analyses. The color bar indicates t values. B1, B2, and B3. Color on the brain images shows regions covered by positive, negative, and neutral ICs, respectively. The color bar indicates number of overlapping ICs.

**Fig 4:**
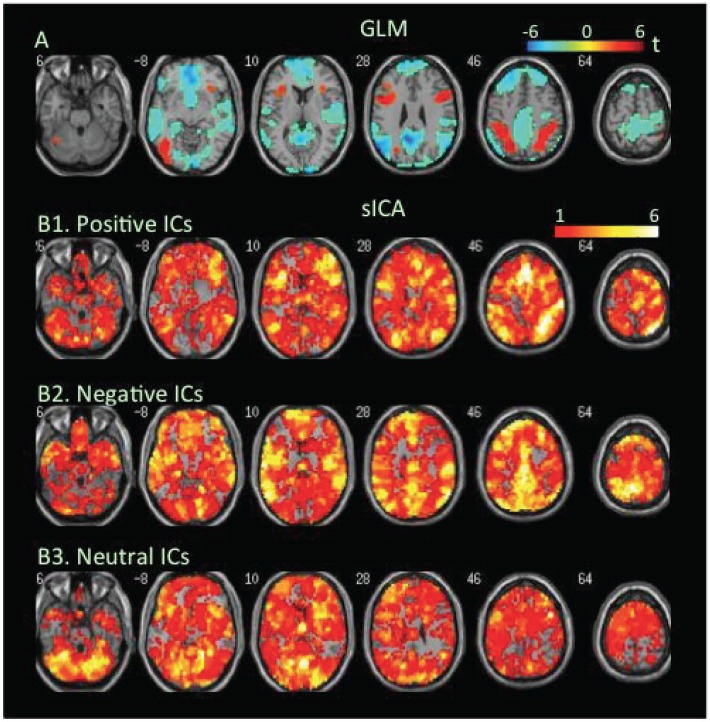
Task-related modulation in BOLD signal during the high-load condition relative to the baseline of the attention task. A. Color on the brain images shows task-related increases and decreases in BOLD signal as revealed by GLM-based analyses. The color bar indicates t values. B1, B2, and B3. Color on the brain images shows regions covered by positive, negative, and neutral ICs, respectively. The color bar indicates number of overlapping ICs.

**Fig 5:**
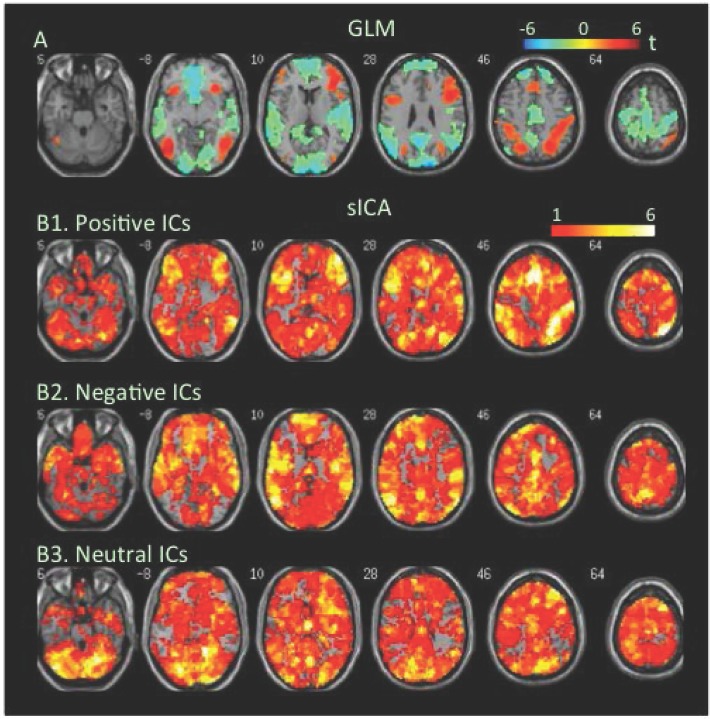
Changes in BOLD signal as task load increased from a low to a high level of the attention task. A. Color on the brain images shows task-related increases and decreases in BOLD signal as revealed by GLM-based analyses. The color bar indicates t values. B1, B2, and B3. Color on the brain images shows regions covered by positive, negative, and neutral ICs, respectively. The color bar indicates number of overlapping ICs.

**Attention task**. An electrophysiological finding of balanced E/I is load-dependent activity increases in cortical neurons accompanied by load-dependent activity decreases in adjacent neurons [73,74]. To assess how task-load-dependent concurrent co-localized increases and decreases in BOLD signal were distributed in the brain, we analyzed changes in BOLD signal at low- and high-load conditions of the attention task. The GLM-BA revealed significant decreases but no significant increases in BOLD signal at low load relative to the resting condition (Fig. 3A, Table 1, Table B in S1 File). During the high-load condition, the GLM-BA revealed further decreases in BOLD signal relative to either resting or low-load condition, and significant increases in BOLD signal in several clusters (Figs. 4A & 5A, Table 1). SICA revealed that positive FNs covered the frontal, parietal, and occipital regions at the low-load condition, while the negative FNs covered even more extensive areas (Fig. 3B1–3). The brain regions covered by positive and/or negative FNs further increased as task load increased from the low- to the high-load condition (Figs. 4B1–3 & 5B1–3, Tables 2 & 3).

**Fig 6:**
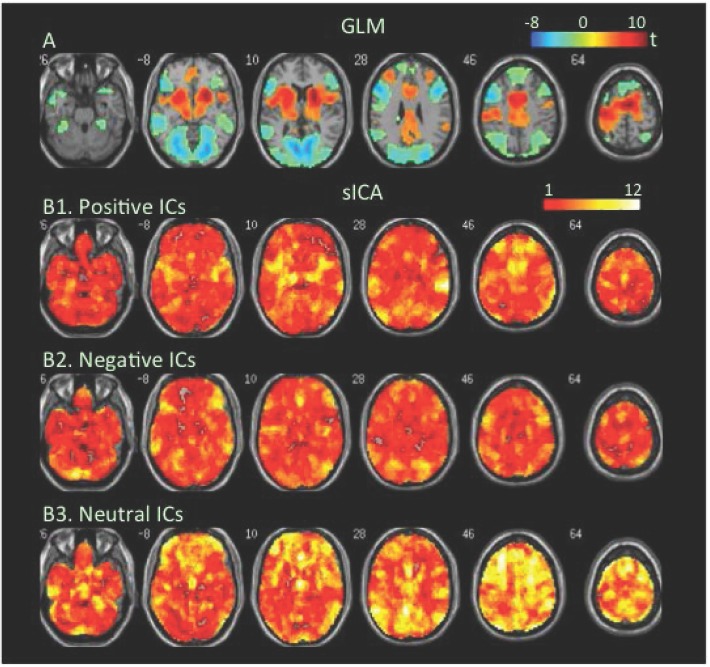
Task-related modulation in BOLD signal at Win $1 relative to the baseline of the MIDT task. A. Color on the brain images shows task-related increases and decreases in BOLD signal as revealed by GLM-based analyses. The color bar indicates t values. B1, B2, and B3. Color on the brain images shows regions covered by positive, negative, and neutral ICs, respectively. The color bar indicates number of overlapping ICs.

**Fig 7:**
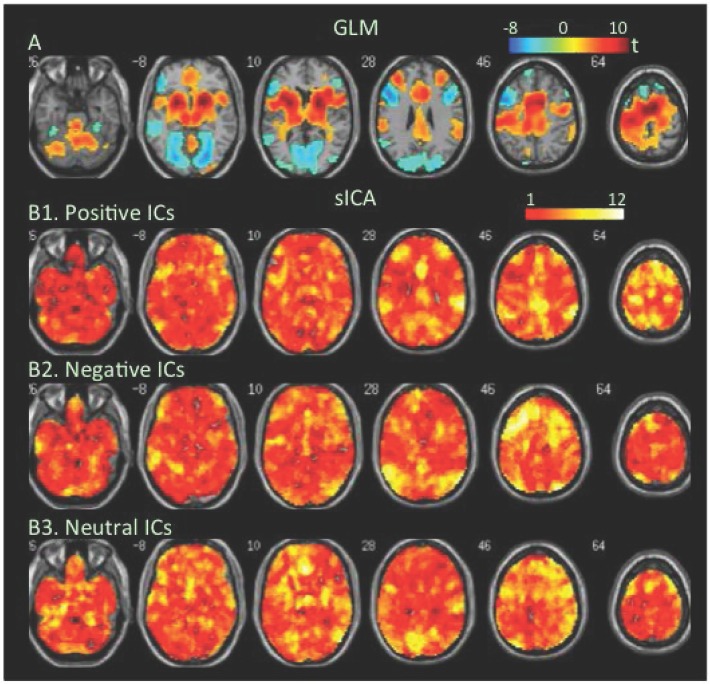
Task-related modulation in BOLD signal at Win $5 relative to the baseline of the MIDT task. A. Color on the brain images shows task-related increases and decreases in BOLD signal as revealed by GLM-based analyses. The color bar indicates t values. B1, B2, and B3. Color on the brain images shows regions covered by positive, negative, and neutral ICs, respectively. The color bar indicates number of overlapping ICs.

**Fig 8:**
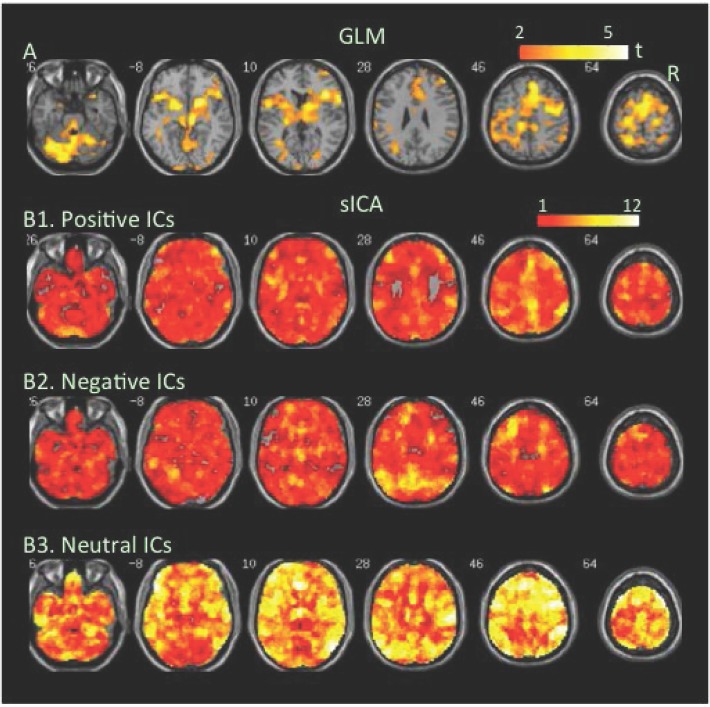
Task-related modulation in BOLD signal as task condition changed from Win $1 to Win $5 of the MIDT task. A. Color on the brain images shows task-related increases and decreases in BOLD signal as revealed by GLM-based analyses. The color bar indicates t values. B1, B2, and B3. Color on the brain images shows regions covered by positive, negative, and neutral ICs, respectively. The color bar indicates number of overlapping ICs.

**MIDT**. This task taps into motivation, attention, and visual perception and imposes two levels reward and punishment. This study focused on the first anticipatory phase (A1) of Win $1 (W1) and Win $5 (W5) trials. The GLM-BA revealed significant increases and decreases in BOLD signal at separated brain regions at W1 relative to control condition (Fig. 6A, Table 1, Table B in S1 File), and more extensive increases but less extensive decreases at W5 relative to either W1 or control condition (Figs. 7A & 8A, Table 1). Noticeably, the cortical regions related to visual perception and attention, such as the parietal and occipital cortex, showed decreases in BOLD signal, even though the MIDT required visual perception and attention. SICA revealed that either positive or negative FNs covered almost the entire brain, including the parietal and occipital cortex, at both W1 and W5 (Figs. 6, 7, 8, Tables 2 & 3).

### Findings across tasks

The positive and negative voxels revealed by the GLM-BA were typically segregated from each other and formed positive and negative clusters, similar to findings of most fMRI studies using a GLM-BA. Most positive clusters overlapped with positive FNs extracted by sICA, indicating that the GLM-BA and sICA identified BOLD signal increases in same brain regions (Table 1). Furthermore, regions showing a greater increase in BOLD signal (i.e., a greater t value) as revealed by the GLM-BA often corresponded to regions showing overlap of more positive FNs (Figs. 1, 2, 3, 4, 5, 6, 7, 8), indicating that these regions were shared by multiple positive FNs. However, the volumes of positive voxels were always smaller than the volumes of positive FNs (Tables 1 & 3). Such relationships between positive voxels and FNs also existed between negative voxels and FNs.

Positive and negative FNs overlapped extensively (Figs. 1, 2, 3, 4, 5, 6, 7, 8, Table 3). The overlap volume was dependent on individual task condition, and ranged from 19% to 95% of the whole brain. More demanding and/or complicated task conditions showed greater overlaps than did less demanding task conditions. The overlap of positive and negative FNs indicates concurrent co-localized increases and decreases in source signals in the same voxels. The non-overlapping positive and negative FNs were not completely segregated into clusters as positive and negative voxels; instead, they intermixed with each other. This mixture of positive and negative FNs indicates concurrent co-localized increases and decreases in source signals in the same brain regions, although not necessarily in the same voxels. The overlaps and mixtures of positive and negative FNs indicate that extensive regions, including both cortical and subcortical structures throughout the brain, may show concurrent co-localized increases and decreases in source signal during the performance of cognitive tasks, especially during complicated or highly demanding tasks.

Most of the volume (38 ∼ 78% of whole brain) of positive and negative FNs did not show significant changes in BOLD signal as assessed by the GLM-BA, indicating that the concurrent co-localized increases and decreases in source signals were equivalent and cancelled each other at these regions. However, the remainder of these regions showed significant increases or decreases in BOLD signal (i.e., regions overlapped with positive or negative voxels revealed by the GLM-BA), indicating that either increases or decreases in source signals dominate over the opposite changes in these regions. Their exact locations were task-dependent as indicated by the GLM-BA. Their volumes ranged from 0% to 53% of the whole brain, and were often smaller than the volumes without significant changes in BOLD signal as revealed by the GLM-BA.

SICA revealed neutral FNs across all task conditions (Figs. 1, 2, 3, 4, 5, 6, 7, 8, Tables 2 & 3). Neutral FNs occupied most or all of the brain (80∼100% of whole brain), and either overlapped or intermixed with both positive and negative FNs. This finding indicates that some neural substrates in each brain region did not show significant changes in activity during task performance; and that these neural substrates were distributed across most of the brain and intermixed with neural substrates showing either increases or decreases in activity.

Relative to the congruent condition of the Flanker task, both the low-load and high-load conditions of the attention task presented a greater demand on perception, attention, and working memory. However, both induced considerably less extensive increases in BOLD signal relative to the congruent condition of the Flanker task, as revealed by the GLM-BA. However, sICA found that both positive and negative FNs covered more extensive brain regions at the high-load condition of the attention task relative to the Flanker task. The fact that these datasets were acquired from different populations using different scanners prevented direct comparison using conventional statistical methods.

### Consistency of ICs within and between datasets

ICASSO found that the stability indices of most ICs were > 0.8 and only 1 to 6 ICs from each dataset showed stability index between 0.6 and 0.8. Therefore, sICA generated stable ICs from each dataset in the current study.

**Fig 9:**
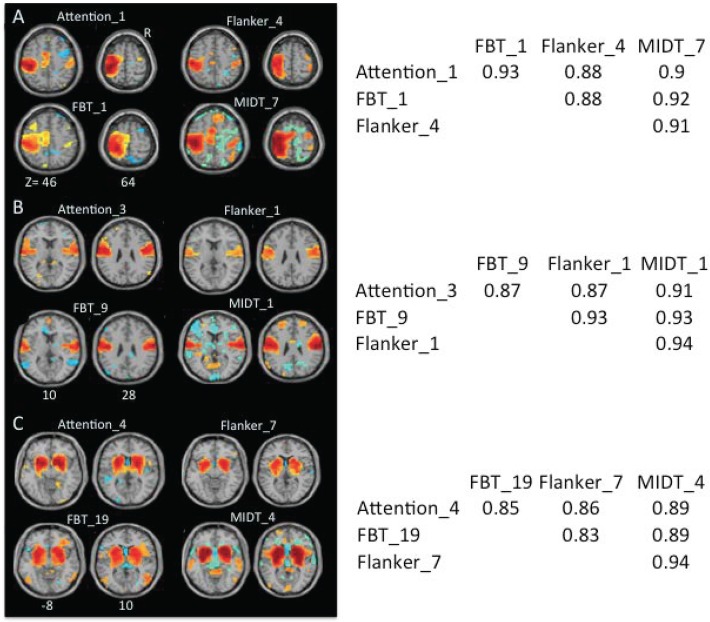
Examples of matched ICs from different datasets. A. Attention_1 shows the first IC from the attention dataset. It matches with Flanker_4, the IC 4 from the Flanker dataset, with FBT_1, the IC 1 from the FBT dataset, and MIDT_7, the IC 7 from the MIDT dataset. The two numbers after z indicate the z coordinates in the MNI space. The table at the right side presents correlation coefficients for each pair of ICs. B and C present matches for IC 3 and IC 4 from the attention dataset, respectively. R indicates the right side of the brain. Please see Fig. B in S1 File for other IC matches.

**Fig 10:**
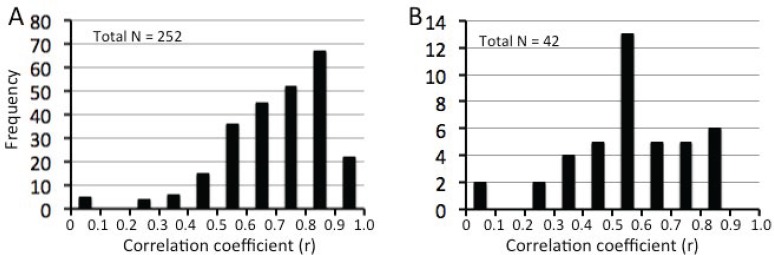
Frequency distributions of correlation coefficients. A shows the frequency distribution of correlation coefficients (X axis) of spatial correlation between each pair of ICs from the four datasets. B. Assessing the spatial correlations of each IC from the attention dataset with ICs from the other three datasets generated six correlation coefficients (Please see the table in Fig. 9). B shows the frequency distribution of the smallest among six correlation coefficients for each IC from the attention dataset.

We identified 42 ICs from the attention dataset as FNs, the lowest number of ICs among the four datasets. Spatial correlation analyses were used to match each of the 42 ICs from the attention dataset to ICs from the other three datasets. This analysis generates six correlation coefficients (r) for each IC from the attention dataset and three matched ICs from other three datasets (Fig. 9), and a total of 252 coefficients for 42 ICs and their matched ICs. Among 252 coefficients, 222 (88.1%) were ≥ 0.5 (Fig. 10). Among 42 ICs from the attention dataset, 13 ICs (31%) showed one or more correlation coefficients < 0.5, while all coefficients for remaining 39 ICs were ≥ 0.5 (Fig. 10). Therefore, most ICs extracted from the four different datasets were highly consistent in spatial patterns (please see Fig. B in S1 File for all ICs showing consistent spatial patterns across the four datasets).

## Discussion

### Overlap of positive, negative, and neutral FNs

SICA extracted multiple positive and negative FNs from each dataset. Consistent with recent findings [8–10,26], the positive FNs overlapped with each other, as did the negative FNs. Furthermore, the positive FNs overlapped with negative FNs extensively. These findings across different tasks and participants in the current and prior studies suggest that task-related, concurrent, co-localized, increases and decreases in source signals (i.e., signals extracted by sICA from BOLD signal mixtures) occur across extensive portions of the brain and are not specific to tasks or participants; rather, the findings suggest that the patterns may reflect a general property of functional brain organization. The spatial extent of concurrent co-localized increases and decreases in the source signals are probably dependent on task-load, because they showed further increases as task load changed from a low to high level during the attention task. The findings indicate that neural substrates showing task-related increases or decreases in activity may intermix with each other across extensive brain regions.

In the current study, the attention task mainly demands central processes related to visual attention. The positive FNs covered about one third of the entire brain at the low-load condition, and about 80% of the whole brain during the high-load condition. Relative to the attention task, the MIDT requires reward anticipation in addition to visual attention and perception. This difference between the two tasks may contribute to the more extensive brain regions covered by the positive and negative FNs related to the low-value condition (W1) of the MIDT than those related to the low-load condition of the attention task. Both the positive and negative FNs related to the MIDT covered almost the entire brain even during W1, and these FNs could not expand to cover more brain regions as task load increased from W1 to W5 due to the ceiling effect. These findings indicate that neural substrates distributed across most of the entire brain are implicated during the performance of high-demand or complex tasks.

Most brain regions showing overlap of task-related positive and negative FNs did not show significant changes in BOLD signal as analyzed using a GLM-BA. These data indicate that the task-related, concurrent, co-localized increases and decreases in source signals were equivalent or balanced and cancelled each other. Even though these regions did not show significant changes in BOLD signal when assessed using a GLM-BA, different source signals within them underwent significant task-related changes as revealed by sICA. These brain regions include both cortical and subcortical areas. Therefore, neural substrates related to positive or negative source signals in these regions were activated or deactivated during task performance, consistent with the notion that sICA may reveal task-related activity which is not detected by the GLM-BA [24].

The GLM-BA in the current study revealed significant changes in BOLD signal in some areas, including those showing overlap of positive and negative FNs. For example, the GLM-BA revealed significant increases in BOLD signal in extensive brain regions but very limited decreases during the congruent condition of the Flanker task, whereas extensive decreases in BOLD signal with no significant increases during the low-load condition of the attention task. Therefore, these brain regions were dominated by either increases or decreases in source signals during different tasks. The exact locations of unbalanced changes in source signals were task-dependent as revealed by the GLM-BA. This finding indicates that not all brain regions maintain a balance of task-related activation and deactivation of neural substrates related to different source signals.

In addition to the positive and negative FNs, sICA revealed multiple “neutral” FNs from each dataset. These “neutral” FNs covered most or all brain regions and overlapped with positive and negative FNs extensively. This finding indicates that some neural substrates related to these “neutral” source signals within each brain regions do not show task-related changes in activity. These “neutral” neural substrates distributed across almost the entire brain and intermixed with neural substrates showing more consistent task-related activation or deactivation.

### Similar and different findings between the GLM-BA and sICA

The GLM-BA and sICA identified common regions showing increases, decreases, or no changes in BOLD signal and source signals, respectively, indicating that the two approaches identified the same brain regions showing consistent changes in BOLD signal and source signals. Besides this similar finding, the two approaches have several important different findings. First, the GLM-BA revealed task-related increases, decreases, and no change in BOLD signals in separated regions without any spatial overlap among them, consistent with most previous findings of GLM-BA. This finding implies that neural substrates showing task-related activation, deactivation, or no changes in activation are segregated and distributed in different brain regions. In comparison, sICA finds extensive spatial overlap among source signals showing increased, decreased, or no changes in activation. These findings suggest that neural substrates showing task-related activation, deactivation, or no changes in activity intermix with each other in the same regions across extensive portion of the brain.

Second, the finding of segregated brain regions showing task-related increases in BOLD signal as revealed by GLM-BA has been interpreted by some investigators as evidence supporting the view of modular processing in the brain [75]. However, sICA found overlap of multiple positive FNs in these “modular regions”. It is possible that FN overlap may facilitate information exchange among synergistic FNs relative to spatially separated FNs. Therefore, these “modular regions” as revealed by the GLM-BA may reflect the activity increases of multiple synergistic FNs. Furthermore, sICA found that positive FNs often covered extensive regions beyond the restricted “modular regions”, indicating a distributed processing in the brain. These findings suggest a hybrid modular and distributed functional organization; i.e., neural substrates with specific functional properties distributed in extensive cortical and subcortical regions with relative higher density at certain regions [50–52].

Third, the GLM-BA revealed activation in the striatum and deactivation in the parietal and occipital cortex during either the W1 or W5 conditions of the MIDT. This finding is consistent with most data from previous reward-anticipation-related fMRI studies using a GLM-BA and task analogs of the MIDT [76]. However, these data are not consistent with the fact that the MIDT involves visual perception and attention and might be expected to activate, not deactivate, parietal and occipital cortex related to visual perception and attention. On the other hand, sICA reveals concurrent co-localized increases and decreases in source signals in these regions, highly consistent with the finding of reward-anticipation-related activation in the right frontoparietal attention network as revealed by sICA in a previous study [76].

Fourth, the GLM-BA reveals significant deactivation without significant activation during the low-load condition of the attention task. It is unlikely that participants can successfully perform the task without activation of any brain regions relative to the resting condition. On the other hand, sICA found extensive increases in source signals in the frontoparietal and occipital cortex accompanied by concurrent co-localized decreases in source signals. The sICA data suggest that the frontoparietal and occipital cortex contribute to task performance, and that the negative finding of the GLM-BA may represent cancellation of opposite changes in source signals. Finally, the GLM-BA reveals a much smaller volume showing increases in BOLD signal (<10%) during the high-load condition of the attention task relative to the congruent condition of the Flanker task, even though the former task condition presents a greater demand on visual perception, attention, and working memory. On the other hand, sICA finds a greater volume showing increases in source signals during the high-load condition relative to the congruent condition, but also a greater volume of decreases in source signals. Therefore, the smaller volume of increases in BOLD signal during the high-load condition as revealed by the GLM-BA may reflect the cancellation of concurrent co-localized increases and decreases in source signals. These different findings suggest that sICA provides additional information related to task-related brain activity relative to the GLM-BA, and further support the view that sICA can reveal task-related activity hidden from GLM-BA [24].

### Consistency in IC spatial maps

Several recent studies assessed the “optimal” model order (i.e., number of ICs) in each dataset and found high-order models involving between 70 and 80 ICs generated refined informative networks corresponding to known anatomical and functional segmentations [63,64,66,67,69,77,78]. Accordingly, this study extracted 75 ICs. ICASSO indicated that most ICs were highly stable (stability indices > 0.8). Spatial correlation analysis revealed that most ICs generated from different datasets were highly consistent in spatial patterns. Each of the four datasets used in the current study was acquired using different scanners at different institutes, while different populations performed different tasks. The high consistency in spatial patterns identified indicates that these ICs likely reflect large-scale patterns of brain activity organization, rather than artifacts arbitrarily generated by the sICA algorithm.

### Potential brain functional properties related to FN overlap

As discussed in the introduction, we hypothesized that balanced E/I and functional heterogeneity might relate to overlap of FNs showing concurrent opposite modulations. In the literature, balanced E/I mainly refers to the close relationship between excitation and inhibition within a neuron and among adjacent neurons in the same brain regions [30–35]. However, fMRI studies using GLM-BA often find concurrent activation and deactivation at different brain regions during either resting condition or task performance [79–81]. For example, cognitive tasks often induce activation in the lateral frontoparietal cortex and concurrent deactivation in the medial frontoparietal cortex, e.g., the default mode network. Therefore, the concurrent activation and deactivation as revealed by GLM-BA probably reflects balanced E/I between different brain regions at the level of large-scale networks.

The balanced E/I and functional heterogeneity combined with the unique capacity of separating signal mixture into source signals of sICA might explain differences in some findings between the GLM-BA and sICA in the current study. For example, the MIDT involved both reward anticipation and visual perception and attention. Reward anticipation induced activation in the striatum, thalamus, insula, and medial PFC, but may induce deactivation in the occipital and parietal cortex because of balanced E/I. The deactivation in these brain regions was probably greater than activation induced by visual perception and attention, and thus the GLM-BA found deactivation. In contrast, sICA revealed concurrent activation and deactivation in these brain regions.

The current study found multiple neutral ICs from each dataset. We hypothesized that they might relate to another brain property, i.e., sparseness of neuron activities [82,83]. Sparseness refers to the property that any neuron is only responsive to a few stimuli among all inputs from the environment (i.e., lifetime sparseness) [84], and that only a small percent of whole neural population in any brain region exhibits activity at any instant (population sparseness), regardless of resting condition or during task performance, while most neurons remain silent [85]. Consistent with this property, electrophysiological studies often find that less than half of all recorded neurons show task-related activity increases [51,52]. Therefore, the current neutral ICs might relate to neural substrates not responsive to task performance. We hypothesized that the extensive overlap of positive, negative, and neutral FNs as revealed by sICA may relate to balanced E/I, functional heterogeneity, and population sparseness. This hypothesis raises a question of whether sICA can separate different source signals associated with neural substrates showing concurrent activation, deactivation, or no changes in activation in the same voxels.

### Methodological consideration and potential limitations

Even though the exact coupling between neural activity and BOLD signal is not clear yet, recent data indicate that BOLD signal increases are related to increased neuronal activity while BOLD signal decreases are related to reduced neuronal activity [86–90]. These studies also found that the neurovascular coupling between reduced neuronal activities and reduced BOLD signal was different from the neurovascular coupling between increased neuronal activity and increased BOLD signal, i.e., the two neurovascular couplings were not mere a reverse of each other [86,89,91]. Therefore, BOLD signal decreases may have different temporal features relative to BOLD signal increases even if their underlying neural activities negatively correlate with each other as stipulated by the property of balanced E/I. Furthermore, inhibitory interneurons and excitatory pyramid neurons connect with adjacent neurons differently. Inhibitory interneurons connect to most adjacent neurons and exert inhibition on adjacent neurons unselectively (i.e., global inhibition or blanket of inhibition) [49,92–94], while excitatory neurons connect to a selected minority of adjacent neurons [95–98]. Therefore, the spatial distributions of activation and deactivation are different at the level of microcircuits. Furthermore, fMRI studies using GLM-BA regularly report task-related increases and decreases in BOLD signal at different brain regions, indicating that they have different distributions in large-scale networks. These data together indicate that concurrent decreases and increases in source signals may have different temporal features and spatial distributions and thus allow sICA to separate them from their mixtures.

One issue related to concurrent increases and decreases in BOLD signal is blood redistribution. The brain may have a limited volume of blood supply, and it is logical to suspect that increases in blood flow in some brain regions may lead to reduced blood flow in other brain regions. While it is not clear the extent of “blood redistribution” contributing to the current finding of concurrent increases and decreases in source signals, some data from this study indicate that it may not be the main factor contributing to source signal reduction as revealed by sICA. For example, GLM-BA revealed extensive BOLD signal increases, but only a very limited decrease during the congruent condition of the Flanker task, and extensive BOLD signal decreases but no significant increase during the low-load condition of the attention task. Therefore, task-related increases and decreases in BOLD signal may not always correlate with each other.

The statistical threshold for defining the spatial extent of each FN may influence the spatial extent of FN overlap. More strict thresholds will lead to a smaller spatial extent of each FN and less spatial overlap of different FNs. Relative to the threshold regularly used in fMRI studies using GLM-BA, the current study used a very strict threshold, i.e., voxel level p <. 001, FDR corrected for multiple comparisons involved in voxel-wise whole brain analysis (equivalent to t > 4.0).

One limitation of this study is that the precise number of ICs existing in each dataset is not known. The number of ICs generated by sICA will likely influence the number of FNs overlapping with each other. Another limitation is that there is no reliable fully objective method available for separating artifacts from real FNs. A third limitation is the uncertainty of an optimal statistical threshold for defining task-positive, task-negative, and task-neutral FNs. Using a strict threshold for positive and negative FNs may lead to a liberal threshold for neutral FNs and vice versa. Therefore, the number of FNs in each category may change depending on the selection of different thresholds. However, the final conclusion of extensive overlap of different FNs still holds at different thresholds.

Finally, we emphasize here, even though this study hypothesizes that balanced E/I, functional heterogeneity, and population sparseness may relate to the extensive overlap of positive, negative, and neutral FNs, the exact relationships or the real neural substrates underlying this finding of sICA remains relatively speculative and should be investigated further in future studies. FN overlap as revealed by fMRI using sICA represents a feature of large-scale networks of brain activity, and the three brain properties (balanced E/I, functional heterogeneity, and population sparseness) represent functional activity at the level of microcircuits. The exact relationships between large-scale networks and microcircuits activity are not clear yet.

In summary, by revealing extensive overlap of positive, negative, and neutral FNs, findings from sICA indicate that neural substrates showing task-related activation, deactivation, and no change in activation intermix with each other and distribute across most or even the whole brain. This pattern of brain functional organization is different from the pattern of separated activation, deactivation, and no-change in activation as often revealed by the GLM-BA. We hypothesized that the extensive overlap of different FNs as revealed by sICA may relate to balanced E/I, functional heterogeneity, and population sparseness, three fundamental properties of the brain, and their exact relationships deserve further investigation.

## Supporting Information

S1 File Fig. A, ICASSO results. Fig. B, Matched ICs from different datasets. Table A, Beta weights at each task condition. Table B, Brain regions showing task-related changes in BOLD signal as assessed by SPM5.
(DOCX)
