# Supplementary material for: Functional Network Overlap as Revealed by fMRI Using sICA and Its Potential Relationships with Functional Heterogeneity, Balanced Excitation and Inhibition, and Sparseness of Neuron Activity
Source: PLoS One. 2015 Feb 25;10(2):e0117029. doi: 10.1371/journal.pone.0117029 (PMC4340936; doi:10.1371/journal.pone.0117029)
Supplement: S1 File — Fig. B, Matched ICs from different datasets. Table A, Beta weights at each task condition. Table B, Brain regions showing task-related changes in BOLD signal as assessed by SPM5. (DOCX) [file pone.0117029.s001.docx]

**Supplementary Materials For**

**Functional network overlap as revealed by fMRI using sICA and its potential relationships with functional heterogeneity, balanced excitation and inhibition, and sparseness of neuron activity**

Jiansong Xu, Vince D. Calhoun, Patrick D. Worhunsky, Hui Xiang, Jia Li, John T. Wall, Godfrey D. Pearlson, Marc N. Potenza

Table A. Beta weights at each task condition.

| **Flanker Task** | | | | | | | | | | | | |
| --- | --- | --- | --- | --- | --- | --- | --- | --- | --- | --- | --- | --- |
| **Positive ICs** | | | | | | | | | | | | |
| IC# | IC4 | IC7 | IC28 | IC29 | IC35 | IC38 | IC43 | IC44 | IC53 | IC54 | IC59 | IC62 |
| mean | 0.648 | 0.167 | 0.217 | 0.229 | 0.224 | 0.183 | 0.247 | 0.261 | 0.234 | 0.159 | 0.184 | 0.270 |
| Raw p | 0.000 | 0.000 | 0.000 | 0.008 | 0.003 | 0.001 | 0.017 | 0.000 | 0.002 | 0.022 | 0.006 | 0.000 |
| FDR p | 0.000 | 0.000 | 0.001 | 0.021 | 0.010 | 0.003 | 0.040 | 0.001 | 0.008 | 0.048 | 0.017 | 0.002 |
| **Positive ICs** | | | | | | | | | | | | |
| IC# | IC65 | IC66 | IC68 | IC74 |  |  |  |  |  |  |  |  |
| mean | 0.262 | 0.149 | 0.524 | 0.572 |  |  |  |  |  |  |  |  |
| Raw p | 0.002 | 0.018 | 0.000 | 0.000 |  |  |  |  |  |  |  |  |
| FDR p | 0.007 | 0.040 | 0.000 | 0.000 |  |  |  |  |  |  |  |  |
|  |  |  |  |  |  |  |  |  |  |  |  |  |
| **Negative ICs** | | | | | | | | | | | | |
| IC# | IC5 | IC6 | IC30 | IC32 | IC58 |  |  |  |  |  |  |  |
| mean | -0.167 | -0.136 | -0.226 | -0.233 | -0.065 |  |  |  |  |  |  |  |
| Raw p | 0.006 | 0.006 | 0.000 | 0.000 | 0.006 |  |  |  |  |  |  |  |
| FDR p | 0.017 | 0.017 | 0.002 | 0.000 | 0.017 |  |  |  |  |  |  |  |
|  |  |  |  |  |  |  |  |  |  |  |  |  |
| **Neutral ICs** | | | | | | | | | | | | |
| IC# | IC1 | IC2 | IC11 | IC12 | IC18 | IC20 | IC22 | IC27 | IC31 | IC36 | IC39 | IC41 |
| mean | 0.006 | -0.108 | -0.092 | 0.011 | -0.043 | -0.135 | 0.097 | -0.072 | -0.036 | -0.047 | 0.037 | -0.086 |
| Raw p | 0.891 | 0.197 | 0.353 | 0.834 | 0.481 | 0.026 | 0.310 | 0.157 | 0.343 | 0.231 | 0.376 | 0.027 |
| FDR p | 0.891 | 0.324 | 0.438 | 0.855 | 0.553 | 0.053 | 0.417 | 0.268 | 0.438 | 0.349 | 0.444 | 0.053 |
| **Neutral ICs** | | | | | | | | | | | | |
| IC# | IC48 | IC50 | IC52 | IC55 | IC45 | IC61 | IC63 | IC64 | IC67 | IC71 | IC72 | IC73 |
| mean | 0.034 | 0.009 | 0.056 | 0.019 | 0.032 | 0.031 | -0.074 | 0.111 | -0.028 | -0.080 | -0.071 | -0.104 |
| Raw p | 0.549 | 0.701 | 0.070 | 0.836 | 0.250 | 0.317 | 0.555 | 0.222 | 0.366 | 0.130 | 0.114 | 0.248 |
| FDR p | 0.608 | 0.750 | 0.134 | 0.855 | 0.349 | 0.417 | 0.608 | 0.349 | 0.443 | 0.231 | 0.209 | 0.349 |
| **Neutral ICs** | | | | | | | | | | | | |
| IC# | IC75 |  |  |  |  |  |  |  |  |  |  |  |
| mean | 0.091 |  |  |  |  |  |  |  |  |  |  |  |
| Raw p | 0.240 |  |  |  |  |  |  |  |  |  |  |  |
| FDR p | 0.349 |  |  |  |  |  |  |  |  |  |  |  |
|  |  |  |  |  |  |  |  |  |  |  |  |  |
| **False Belief Task** | | | | | | | | | | | | |
| **Positive ICs** | | | | | | | | | | | | |
| IC# | IC9 | IC12 | IC17 | IC26 | IC30 | IC32 | IC38 | IC40 | IC42 | IC51 | IC59 | IC62 |
| mean | 0.085 | 0.338 | 0.179 | 0.196 | 0.173 | 0.796 | 0.848 | 1.121 | 0.182 | 0.228 | 0.192 | 0.418 |
| Raw p | 0.000 | 0.000 | 0.002 | 0.000 | 0.001 | 0.000 | 0.000 | 0.000 | 0.001 | 0.000 | 0.000 | 0.000 |
| FDR p | 0.000 | 0.000 | 0.003 | 0.000 | 0.001 | 0.000 | 0.000 | 0.000 | 0.002 | 0.001 | 0.000 | 0.000 |
| **Positive ICs** | | | | | | | | | | | | |
| IC# | IC70 |  |  |  |  |  |  |  |  |  |  |  |
| mean | 0.126 |  |  |  |  |  |  |  |  |  |  |  |
| Raw p | 0.001 |  |  |  |  |  |  |  |  |  |  |  |
| FDR p | 0.001 |  |  |  |  |  |  |  |  |  |  |  |
|  |  |  |  |  |  |  |  |  |  |  |  |  |
| **Negative ICs** | | | | | | | | | | | | |
| IC# | IC1 | IC2 | IC11 | IC23 | IC25 | IC39 | IC47 | IC48 | IC49 | IC50 | IC52 | IC57 |
| mean | -0.282 | -0.169 | -0.064 | -0.369 | -0.424 | -0.206 | -0.092 | -0.200 | -0.325 | -0.251 | -0.127 | -0.174 |
| Raw p | 0.000 | 0.000 | 0.025 | 0.025 | 0.000 | 0.000 | 0.001 | 0.000 | 0.000 | 0.000 | 0.005 | 0.000 |
| FDR p | 0.000 | 0.000 | 0.041 | 0.041 | 0.000 | 0.000 | 0.001 | 0.000 | 0.000 | 0.000 | 0.008 | 0.001 |
| **Negative ICs** | | | | | | | | | | | | |
| IC# | IC58 | IC64 | IC66 | IC73 |  |  |  |  |  |  |  |  |
| mean | -0.244 | -0.104 | -0.394 | -0.207 |  |  |  |  |  |  |  |  |
| Raw p | 0.000 | 0.000 | 0.000 | 0.000 |  |  |  |  |  |  |  |  |
| FDR p | 0.000 | 0.000 | 0.000 | 0.000 |  |  |  |  |  |  |  |  |
|  |  |  |  |  |  |  |  |  |  |  |  |  |
| **Neutral ICs** | | | | | | | | | | | | |
| IC# | IC16 | IC19 | IC27 | IC28 | IC33 | IC34 | IC35 | IC37 | IC4 | IC43 | IC44 | IC53 |
| mean | -0.143 | -0.024 | -0.029 | 0.137 | 0.010 | 0.056 | 0.124 | -0.043 | -0.058 | -0.121 | 0.081 | 0.024 |
| Raw p | 0.033 | 0.272 | 0.694 | 0.102 | 0.824 | 0.250 | 0.041 | 0.155 | 0.498 | 0.034 | 0.077 | 0.463 |
| FDR p | 0.051 | 0.320 | 0.725 | 0.129 | 0.841 | 0.301 | 0.060 | 0.192 | 0.532 | 0.051 | 0.100 | 0.518 |
| **Neutral ICs** | | | | | | | | | | | | |
| IC# | IC54 | IC60 | IC61 | IC71 | IC72 | IC74 |  |  |  |  |  |  |
| mean | -0.093 | 0.058 | -0.054 | -0.014 | -0.002 | -0.046 |  |  |  |  |  |  |
| Raw p | 0.073 | 0.060 | 0.071 | 0.478 | 0.966 | 0.403 |  |  |  |  |  |  |
| FDR p | 0.097 | 0.085 | 0.097 | 0.522 | 0.966 | 0.462 |  |  |  |  |  |  |
|  |  |  |  |  |  |  |  |  |  |  |  |  |
| **Attention Task** | | | | | | | | | | | | |
| **Low Load Condition (L1)** | | | | | | | | | | | | |
| **Positive ICs** | | | | | | | | | | | | |
| IC# | IC12 |  |  |  |  |  |  |  |  |  |  |  |
| mean | 0.187 |  |  |  |  |  |  |  |  |  |  |  |
| Raw p | 0.000 |  |  |  |  |  |  |  |  |  |  |  |
| FDR p | 0.000 |  |  |  |  |  |  |  |  |  |  |  |
|  |  |  |  |  |  |  |  |  |  |  |  |  |
| **Negative ICs** | | | | | | | | | | | | |
| IC# | IC36 | IC47 | IC51 | IC54 | IC63 | IC65 | IC66 | IC67 | IC74 |  |  |  |
| mean | -0.190 | -0.067 | -0.159 | -0.147 | -0.155 | -0.076 | -0.204 | -0.094 | -0.192 |  |  |  |
| Raw p | 0.002 | 0.002 | 0.000 | 0.012 | 0.003 | 0.001 | 0.004 | 0.000 | 0.005 |  |  |  |
| FDR p | 0.014 | 0.013 | 0.003 | 0.048 | 0.019 | 0.008 | 0.022 | 0.000 | 0.022 |  |  |  |
|  |  |  |  |  |  |  |  |  |  |  |  |  |
| **Neutral ICs** | | | | | | | | | | | | |
| IC# | IC1 | IC3 | IC4 | IC10 | IC14 | IC15 | IC17 | IC18 | IC19 | IC20 | IC21 | IC22 |
| mean | 0.022 | 0.006 | 0.025 | -0.053 | 0.024 | -0.085 | 0.019 | 0.000 | -0.113 | 0.021 | -0.073 | -0.034 |
| Raw p | 0.250 | 0.676 | 0.077 | 0.125 | 0.215 | 0.057 | 0.275 | 0.997 | 0.065 | 0.254 | 0.071 | 0.219 |
| FDR p | 0.411 | 0.811 | 0.170 | 0.262 | 0.383 | 0.165 | 0.428 | 0.997 | 0.165 | 0.411 | 0.165 | 0.383 |
| **Neutral ICs** | | | | | | | | | | | | |
| IC# | IC24 | IC27 | IC29 | IC31 | IC35 | IC37 | IC41 | IC42 | IC43 | IC45 | IC49 | IC53 |
| mean | 0.004 | -0.101 | 0.070 | -0.066 | -0.059 | -0.067 | -0.012 | 0.014 | 0.001 | 0.036 | 0.020 | -0.011 |
| Raw p | 0.868 | 0.026 | 0.055 | 0.153 | 0.356 | 0.025 | 0.698 | 0.403 | 0.956 | 0.070 | 0.391 | 0.483 |
| FDR p | 0.911 | 0.092 | 0.165 | 0.306 | 0.535 | 0.092 | 0.814 | 0.546 | 0.980 | 0.165 | 0.546 | 0.629 |
| **Neutral ICs** | | | | | | | | | | | | |
| IC# | IC55 | IC56 | IC58 | IC59 | IC60 | IC62 | IC68 | IC73 |  |  |  |  |
| mean | 0.012 | 0.014 | 0.016 | 0.024 | 0.026 | -0.004 | 0.013 | -0.092 |  |  |  |  |
| Raw p | 0.513 | 0.494 | 0.742 | 0.203 | 0.371 | 0.778 | 0.756 | 0.066 |  |  |  |  |
| FDR p | 0.634 | 0.629 | 0.836 | 0.383 | 0.537 | 0.838 | 0.836 | 0.165 |  |  |  |  |
|  |  |  |  |  |  |  |  |  |  |  |  |  |
| **High Load Condition (L3)** | | | | | | | | | | | | |
| **Positive ICs** | | | | | | | | | | | | |
| IC# | IC19 | IC20 | IC22 | IC27 | IC37 | IC63 | IC74 |  |  |  |  |  |
| mean | 0.380 | 0.126 | 0.059 | 0.328 | 0.085 | 0.249 | 0.182 |  |  |  |  |  |
| Raw p | 0.000 | 0.000 | 0.003 | 0.000 | 0.000 | 0.000 | 0.001 |  |  |  |  |  |
| FDR p | 0.000 | 0.001 | 0.005 | 0.000 | 0.000 | 0.000 | 0.002 |  |  |  |  |  |
|  |  |  |  |  |  |  |  |  |  |  |  |  |
| **Negative ICs** | | | | | | | | | | | | |
| IC# | IC3 | IC10 | IC14 | IC15 | IC18 | IC21 | IC24 | IC36 | IC41 | IC43 | IC49 | IC51 |
| mean | -0.063 | -0.315 | -0.100 | -0.228 | -0.203 | -0.151 | -0.223 | -0.180 | -0.225 | -0.202 | -0.116 | -0.197 |
| Raw p | 0.001 | 0.001 | 0.000 | 0.000 | 0.000 | 0.000 | 0.000 | 0.020 | 0.000 | 0.000 | 0.000 | 0.000 |
| FDR p | 0.002 | 0.001 | 0.001 | 0.001 | 0.000 | 0.000 | 0.000 | 0.030 | 0.000 | 0.000 | 0.000 | 0.000 |
| **Negative ICs** | | | | | | | | | | | | |
| IC# | IC53 | IC55 | IC56 | IC58 | IC65 | IC66 | IC67 | IC68 | IC73 |  |  |  |
| mean | -0.210 | -0.065 | -0.133 | -0.513 | -0.237 | -0.217 | -0.175 | -0.352 | -0.455 |  |  |  |
| Raw p | 0.000 | 0.002 | 0.000 | 0.000 | 0.000 | 0.000 | 0.008 | 0.000 | 0.000 |  |  |  |
| FDR p | 0.000 | 0.004 | 0.000 | 0.000 | 0.000 | 0.001 | 0.012 | 0.000 | 0.000 |  |  |  |
|  |  |  |  |  |  |  |  |  |  |  |  |  |
| **Neutral ICs** | | | | | | | | | | | | |
| IC# | IC1 | IC4 | IC12 | IC17 | IC29 | IC31 | IC35 | IC42 | IC45 | IC47 | IC54 | IC59 |
| mean | -0.048 | 0.026 | 0.003 | 0.013 | 0.008 | -0.118 | -0.045 | 0.007 | -0.037 | 0.033 | -0.063 | -0.004 |
| Raw p | 0.085 | 0.146 | 0.887 | 0.552 | 0.844 | 0.115 | 0.443 | 0.616 | 0.114 | 0.262 | 0.123 | 0.921 |
| FDR p | 0.124 | 0.181 | 0.908 | 0.610 | 0.886 | 0.151 | 0.503 | 0.663 | 0.151 | 0.315 | 0.156 | 0.921 |
| **Neutral ICs** | | | | | | | | | | | | |
| IC# | IC60 | IC62 |  |  |  |  |  |  |  |  |  |  |
| mean | -0.027 | -0.033 |  |  |  |  |  |  |  |  |  |  |
| Raw p | 0.433 | 0.096 |  |  |  |  |  |  |  |  |  |  |
| FDR p | 0.503 | 0.134 |  |  |  |  |  |  |  |  |  |  |
|  |  |  |  |  |  |  |  |  |  |  |  |  |
| **Low vs. High Load Condition (L3-L1)** | | | | | | | | | | | | |
| **Positive ICs** | | | | | | | | | | | | |
| IC# | IC19 | IC20 | IC22 | IC27 | IC37 | IC47 | IC63 | IC74 |  |  |  |  |
| Raw p | 0.000 | 0.001 | 0.004 | 0.000 | 0.000 | 0.013 | 0.000 | 0.001 |  |  |  |  |
| FDR p | 0.034 | 0.006 | 0.002 | 0.025 | 0.003 | 0.001 | 0.023 | 0.008 |  |  |  |  |
|  |  |  |  |  |  |  |  |  |  |  |  |  |
| **Negative ICs** | | | | | | | | | | | | |
| IC# | IC3 | IC10 | IC12 | IC14 | IC18 | IC24 | IC41 | IC43 | IC45 | IC49 | IC53 | IC55 |
| Raw p | 0.000 | 0.002 | 0.000 | 0.003 | 0.000 | 0.000 | 0.000 | 0.000 | 0.014 | 0.000 | 0.000 | 0.020 |
| FDR p | 0.000 | 0.000 | 0.001 | 0.000 | 0.000 | 0.000 | 0.000 | 0.000 | 0.001 | 0.000 | 0.000 | 0.000 |
| **Negative ICs** | | | | | | | | | | | | |
| IC# | IC56 | IC58 | IC65 | IC68 | IC73 |  |  |  |  |  |  |  |
| Raw p | 0.000 | 0.000 | 0.000 | 0.000 | 0.000 |  |  |  |  |  |  |  |
| FDR p | 0.000 | 0.000 | 0.000 | 0.000 | 0.000 |  |  |  |  |  |  |  |
|  |  |  |  |  |  |  |  |  |  |  |  |  |
| **Neutral ICs** | | | | | | | | | | | | |
| IC# | IC1 | IC4 | IC15 | IC17 | IC21 | IC29 | IC31 | IC35 | IC36 | IC42 | IC51 | IC54 |
| Raw p | 0.033 | 0.973 | 0.033 | 0.847 | 0.101 | 0.240 | 0.632 | 0.888 | 0.917 | 0.742 | 0.427 | 0.276 |
| FDR p | 0.052 | 0.973 | 0.052 | 0.923 | 0.146 | 0.326 | 0.737 | 0.932 | 0.939 | 0.842 | 0.528 | 0.362 |
| **Neutral ICs** | | | | | | | | | | | | |
| IC# | IC59 | IC60 | IC62 | IC66 | IC67 |  |  |  |  |  |  |  |
| Raw p | 0.557 | 0.286 | 0.077 | 0.857 | 0.232 |  |  |  |  |  |  |  |
| FDR p | 0.668 | 0.363 | 0.115 | 0.923 | 0.325 |  |  |  |  |  |  |  |
|  |  |  |  |  |  |  |  |  |  |  |  |  |
| **MIDT** | | | | | | | | | | | | |
| **Win $1 (W1)** | | | | | | | | | | | | |
| **Positive ICs** | | | | | | | | | | | | |
| IC# | IC4 | IC7 | IC14 | IC25 | IC35 | IC44 | IC60 | IC63 | IC67 |  |  |  |
| mean | 0.104 | 0.215 | 0.102 | 0.046 | 0.167 | 0.034 | 0.049 | 0.282 | 0.074 |  |  |  |
| Raw p | 0.000 | 0.000 | 0.000 | 0.025 | 0.001 | 0.016 | 0.002 | 0.000 | 0.000 |  |  |  |
| FDR p | 0.000 | 0.000 | 0.000 | 0.045 | 0.002 | 0.030 | 0.004 | 0.000 | 0.000 |  |  |  |
|  |  |  |  |  |  |  |  |  |  |  |  |  |
| **Negative ICs** | | | | | | | | | | | | |
| IC# | IC11 | IC12 | IC33 | IC38 | IC39 | IC43 | IC50 | IC51 | IC53 | IC55 | IC56 | IC58 |
| mean | -0.116 | -0.219 | -0.111 | -0.159 | -0.090 | -0.069 | -0.192 | -0.075 | -0.071 | -0.062 | -0.093 | -0.051 |
| Raw p | 0.003 | 0.004 | 0.000 | 0.000 | 0.000 | 0.004 | 0.000 | 0.000 | 0.008 | 0.000 | 0.011 | 0.006 |
| FDR p | 0.008 | 0.010 | 0.000 | 0.000 | 0.000 | 0.009 | 0.000 | 0.000 | 0.016 | 0.001 | 0.021 | 0.013 |
| **Negative ICs** | | | | | | | | | | | | |
| IC# | IC61 | IC65 | IC71 |  |  |  |  |  |  |  |  |  |
| mean | -0.098 | -0.060 | -0.045 |  |  |  |  |  |  |  |  |  |
| Raw p | 0.000 | 0.000 | 0.000 |  |  |  |  |  |  |  |  |  |
| FDR p | 0.000 | 0.001 | 0.001 |  |  |  |  |  |  |  |  |  |
|  |  |  |  |  |  |  |  |  |  |  |  |  |
| **Neutral ICs** | | | | | | | | | | | | |
| IC# | IC1 | IC8 | IC9 | IC20 | IC30 | IC31 | IC37 | IC40 | IC41 | IC42 | IC46 | IC47 |
| mean | -0.004 | -0.009 | 0.054 | -0.036 | 0.046 | 0.004 | 0.063 | -0.001 | 0.000 | -0.012 | 0.032 | 0.078 |
| Raw p | 0.728 | 0.510 | 0.163 | 0.048 | 0.031 | 0.808 | 0.098 | 0.981 | 0.986 | 0.831 | 0.423 | 0.070 |
| FDR p | 0.843 | 0.624 | 0.239 | 0.081 | 0.055 | 0.891 | 0.149 | 0.986 | 0.986 | 0.891 | 0.532 | 0.114 |
| **Neutral ICs** | | | | | | | | | | | | |
| IC# | IC52 | IC54 | IC59 | IC62 | IC64 | IC66 | IC70 | IC72 |  |  |  |  |
| mean | 0.045 | -0.001 | 0.033 | 0.027 | 0.009 | -0.030 | -0.008 | 0.006 |  |  |  |  |
| Raw p | 0.177 | 0.951 | 0.293 | 0.085 | 0.556 | 0.336 | 0.341 | 0.811 |  |  |  |  |
| FDR p | 0.252 | 0.986 | 0.402 | 0.134 | 0.662 | 0.441 | 0.441 | 0.891 |  |  |  |  |
|  |  |  |  |  |  |  |  |  |  |  |  |  |
| **Win $5 (W5)** | | | | | | | | | | | | |
| **Positive ICs** | | | | | | | | | | | | |
| IC# | IC4 | IC7 | IC11 | IC12 | IC25 | IC30 | IC31 | IC37 | IC40 | IC41 | IC44 | IC47 |
| mean | 0.204 | 0.381 | 0.270 | 0.339 | 0.191 | 0.093 | 0.064 | 0.092 | 0.151 | 0.081 | 0.071 | 0.266 |
| Raw p | 0.000 | 0.000 | 0.000 | 0.001 | 0.000 | 0.000 | 0.005 | 0.012 | 0.001 | 0.023 | 0.000 | 0.000 |
| FDR p | 0.000 | 0.000 | 0.000 | 0.004 | 0.000 | 0.000 | 0.009 | 0.020 | 0.002 | 0.039 | 0.001 | 0.000 |
| **Negative ICs** | | | | | | | | | | | | |
| IC# | IC53 | IC60 | IC62 | IC63 | IC67 | IC70 | IC72 |  |  |  |  |  |
| mean | 0.116 | 0.125 | 0.109 | 0.481 | 0.161 | 0.034 | 0.125 |  |  |  |  |  |
| Raw p | 0.003 | 0.000 | 0.000 | 0.000 | 0.000 | 0.005 | 0.000 |  |  |  |  |  |
| FDR p | 0.007 | 0.000 | 0.000 | 0.000 | 0.000 | 0.009 | 0.000 |  |  |  |  |  |
|  |  |  |  |  |  |  |  |  |  |  |  |  |
| **Negative ICs** | | | | | | | | | | | | |
| IC# | IC33 | IC39 | IC43 | IC54 | IC55 | IC61 | IC65 | IC66 |  |  |  |  |
| mean | -0.110 | -0.071 | -0.104 | -0.102 | -0.040 | -0.057 | -0.056 | -0.073 |  |  |  |  |
| Raw p | 0.000 | 0.005 | 0.000 | 0.000 | 0.005 | 0.002 | 0.004 | 0.028 |  |  |  |  |
| FDR p | 0.000 | 0.009 | 0.001 | 0.000 | 0.009 | 0.004 | 0.009 | 0.045 |  |  |  |  |
|  |  |  |  |  |  |  |  |  |  |  |  |  |
| **Neutral ICs** | | | | | | | | | | | | |
| IC# | IC1 | IC8 | IC9 | IC14 | IC20 | IC35 | IC38 | IC42 | IC46 | IC50 | IC51 | IC52 |
| mean | -0.033 | -0.011 | -0.037 | 0.043 | 0.024 | 0.007 | -0.013 | -0.011 | 0.059 | 0.042 | -0.018 | -0.003 |
| Raw p | 0.095 | 0.707 | 0.404 | 0.086 | 0.313 | 0.870 | 0.662 | 0.888 | 0.166 | 0.438 | 0.414 | 0.953 |
| FDR p | 0.144 | 0.797 | 0.520 | 0.136 | 0.431 | 0.909 | 0.767 | 0.909 | 0.244 | 0.535 | 0.520 | 0.953 |
| **Neutral ICs** | | | | | | | | | | | | |
| IC# | IC56 | IC58 | IC59 | IC64 | IC71 |  |  |  |  |  |  |  |
| mean | 0.031 | -0.010 | 0.066 | -0.004 | -0.004 |  |  |  |  |  |  |  |
| Raw p | 0.390 | 0.580 | 0.264 | 0.835 | 0.841 |  |  |  |  |  |  |  |
| FDR p | 0.520 | 0.690 | 0.374 | 0.902 | 0.902 |  |  |  |  |  |  |  |
|  |  |  |  |  |  |  |  |  |  |  |  |  |
| **Win$5 vs. Win$1 (W5-W1)** | | | | | | | | | | | | |
| **Positive ICs** | | | | | | | | | | | | |
| IC# | IC4 | IC7 | IC11 | IC12 | IC25 | IC30 | IC31 | IC40 | IC47 | IC50 | IC53 | IC56 |
| Raw p | 0.000 | 0.000 | 0.000 | 0.000 | 0.001 | 0.013 | 0.017 | 0.004 | 0.008 | 0.000 | 0.000 | 0.009 |
| FDR p | 0.000 | 0.001 | 0.000 | 0.000 | 0.003 | 0.029 | 0.036 | 0.012 | 0.021 | 0.000 | 0.000 | 0.022 |
| **Positive ICs** | | | | | | | | | | | | |
| IC# | IC60 | IC62 | IC63 | IC67 | IC70 | IC72 |  |  |  |  |  |  |
| Raw p | 0.000 | 0.008 | 0.000 | 0.004 | 0.009 | 0.003 |  |  |  |  |  |  |
| FDR p | 0.002 | 0.022 | 0.001 | 0.012 | 0.022 | 0.010 |  |  |  |  |  |  |
|  |  |  |  |  |  |  |  |  |  |  |  |  |
| **Negative ICs** | | | | | | | | | | | | |
| IC# | IC35 | IC38 | IC54 | IC71 |  |  |  |  |  |  |  |  |
| Raw p | 0.004 | 0.000 | 0.000 | 0.036 |  |  |  |  |  |  |  |  |
| FDR p | 0.012 | 0.000 | 0.001 | 0.045 |  |  |  |  |  |  |  |  |
|  |  |  |  |  |  |  |  |  |  |  |  |  |
| **Neutral ICs** | | | | | | | | | | | | |
| IC# | IC1 | IC8 | IC9 | IC14 | IC20 | IC33 | IC37 | IC39 | IC41 | IC42 | IC43 | IC44 |
| Raw p | 0.263 | 0.951 | 0.166 | 0.088 | 0.070 | 0.989 | 0.505 | 0.614 | 0.053 | 0.992 | 0.323 | 0.134 |
| FDR p | 0.374 | 0.992 | 0.244 | 0.149 | 0.123 | 0.992 | 0.617 | 0.711 | 0.101 | 0.992 | 0.431 | 0.210 |
| **Neutral ICs** | | | | | | | | | | | | |
| IC# | IC46 | IC51 | IC52 | IC55 | IC58 | IC59 | IC61 | IC64 | IC65 | IC66 |  |  |
| Raw p | 0.706 | 0.061 | 0.348 | 0.292 | 0.156 | 0.563 | 0.100 | 0.643 | 0.864 | 0.341 |  |  |
| FDR p | 0.777 | 0.111 | 0.438 | 0.402 | 0.236 | 0.669 | 0.164 | 0.726 | 0.927 | 0.438 |  |  |

Raw p: p value before correction for multiple comparisons; FDR p: p value after FDR correction

Table B. Brain regions showing task-related changes in BOLD signal as assessed by SPM5

| Regions | L/R | BA | Size(voxels) | Z-Value | MNI Coordinates | | |
| --- | --- | --- | --- | --- | --- | --- | --- |
|  |  |  |  |  | X | Y | Z |
| **Flanker Task** – congruent condition, positive cluster | | | | | | | |
| SFG, MFG, IFG,SMA, ACC, PCC, Superior & Middle & Inferior Temporal G,Superior & Inferior Parietal Lobe, Superior & Middle & Inferior Occipital G, Insula, Thalamus, Caudate, Putamen, Cerebellum anterior & posterior lobe | L/R | 2, 3, 4, 6, 7, 13, 18, 19, 22, 24, 3132, 37, 40, 47 | 31873 | 7.5 | 45 | -66 | -15 |
| **Flanker Task** – congruent condition, negative cluster | | | | | | | |
| Medial SFG & OFC | L/R | 10 | 493 | 3.7 | 3 | 60 | 24 |
| **FBT –** reading false belief script, positive clusters | | | | | | | |
| SFG, MFG, IFG, Superior & Middle & Inferior Temporal G,Superior & Middle & Inferior Occipital G,Precuneus, PCC, Cerebellum Thalamus, Putamen, Putamen | L/R | 6, 17, 18, 19, 20, 21, 22, 38, 30, | 19464 | 7.41 | 15 | -93 | -3 |
| **FBT –** reading false belief script, negative clusters | | | | | | | |
| Inferior Parietal Lobule | L | 40 | 1776 | -4.96 | -54 | -33 | 39 |
| Medial SFG, ACC | L/R | 10, 32 | 2610 | -4.84 | -15 | 51 | 6 |
| Inferior Parietal Lobule | R | 40 | 1006 | -4.76 | 63 | -24 | 33 |
| **Attention task** – low load, negative clusters | | | | | | | |
| IFG | L | 47 | 3098 | 4.35 | -48 | 27 | -15 |
| Medial SFG | R | 8, 10 | 465 | 3.52 | 36 | 42 | -12 |
| PCC | L/R | 31 | 1884 | 3.46 | -6 | -57 | 24 |
| Inferior Parietal Lobe | L | 39 | 478 | 3.39 | -48 | -78 | 30 |
| **Attention task** – high load, positive clusters | | | | | | | |
| Inferior Parietal Lobule, Precuneus | L | 7, 40 | 1254 | 4.34 | -24 | -66 | 42 |
| Inferior Parietal Lobule, Precuneus | R | 7, 40 | 1014 | 3.99 | 45 | -39 | 48 |
| **Attention task** – high load, negative clusters | | | | | | | |
| Precuneus, PCC, Medial SFG | L/R | 5, 6, 9, 10, 31, 32, | 11834 | 4.97 | -6 | -57 | 27 |
| Lingual G | L/R | 17 | 775 | 4.47 | 9 | -105 | 9 |
| **Attention task** – high load > low load | | | | | | | |
| MFG, IFG | R | 46 | 1716 | 5.47 | 45 | 36 | 15 |
| Middle Occipital G | L | 19 | 457 | 4.7 | -45 | -69 | -12 |
| Precuneus, Inferior Parietal Lobe | R | 7, 40 | 994 | 4.64 | 21 | -63 | 48 |
| Middle Occipital G | R | 37 | 398 | 4.47 | 48 | -63 | -15 |
| Precuneus, Inferior Parietal Lobe | L | 7, 40 | 618 | 3.83 | -24 | -69 | 36 |
| **Attention task** – low load > high load | | | | | | | |
| Middle Occipital G, Lingual Gyrus | L/R | 18, 19 | 4721 | 5.78 | 15 | -96 | 30 |
| Superior & Middle Temporal G | L/R | 21, 39, 42 | 2410 | 5.26 | 57 | 3 | -21 |
| Medial SFG & OFC | L/R | 8, 24, 31 | 2652 | 5.23 | -6 | 27 | -18 |
| Superior & Middle Temporal G | L | 21, 22 | 2058 | 4.75 | -66 | -45 | 18 |
| **MIDT –** W1, positive cluster | | | | | | | |
| Medial Frontal Gyrus, SMA, SFG | L/R | 6, 24, 32 | 4057 | Inf | -39 | -18 | 54 |
| Insula, Thalamus, Putamen, Caudate | L/R | 13 | 3621 | Inf | 21 | 12 | 9 |
| **MIDT –** W1, negative cluster | | | | | | | |
| Lingual Gyrus, Middle Occipital G,  Superior & Inferior Parietal Lobe, Precuneus | L/R | 7, 17, 18, 19, 30, 40 | 9665 | Inf | -18 | -66 | 3 |
| MFG, IFG | R | 9, 46 | 2416 | 6.96 | 45 | 15 | 27 |
| **MIDT-** W5, positive cluster | | | | | | | |
| SFG, Medial Frontal Gyrus, ACC, SMA, Precentral Gyrus, Postcentral Gyrus, Insula, Putamen, Thalamus, Caudate, Superior Temporal G, Inferior Parietal Lobe | L/R | 3, 4, 6, 13, 24, 31, 32, 40 | 15876 | Inf | -3 | -6 | 57 |
| Inferior Parietal Lobe | R | 40 | 413 | 4.83 | 57 | -39 | 33 |
| **MIDT-** W5, negative cluster | | | | | | | |
| MFG, IFG | L | 6, 8, 9, 45, 46, 47 | 1077 | 6.63 | -51 | 24 | 21 |
| Lingual G | L/R | 18, 19 | 3964 | 6.14 | 27 | -75 | -9 |
| MFG & IFG | R | 9, 46 | 490 | 5.80 | 48 | 15 | 30 |
| Superior & Middle Temporal G. | L | 21, 22 | 487 | 5.11 | -54 | -39 | -3 |
| **MIDT-** W5 > W1 | | | | | | | |
| SFG, MFG, IFG, SMA, Insula,  Parietal Cortex, Cerebellum, Putamen,  Caudate, Thalamus | L/R | 6, 7, 24, 32, 40 | 11318 | 5.12 | 18 | 9 | -3 |

The MNI (Montreal Neurological Institute) coordinates show the coordinates of the peak voxels. **^†^** The voxel number indicates total brain volume of the cluster showing significant changes in BOLD signal. Abbreviations: ACC: anterior cingulate cortex; G: gyrus; IFG: inferior frontal gyrus; L: left; MFG: middle frontal gyrus; OFC: orbitofrontal cortex; PCC: posterior cingulate cortex; R: right; SFG: superior frontal gyrus; SMA: supplementary motor area. ,

Figure A

**
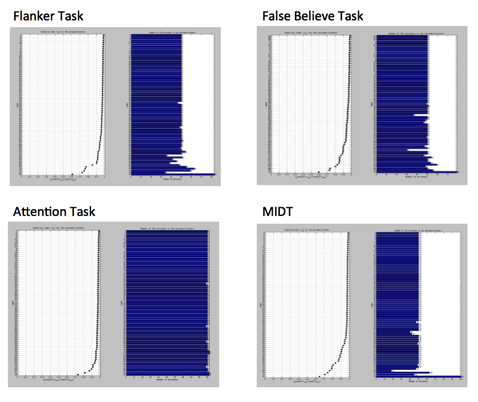
**

Fig. A: ICASSO results. Stability quality index (Iq) and number of ICA estimates in the estimate-clusters of four different tasks.

Figure B

1.


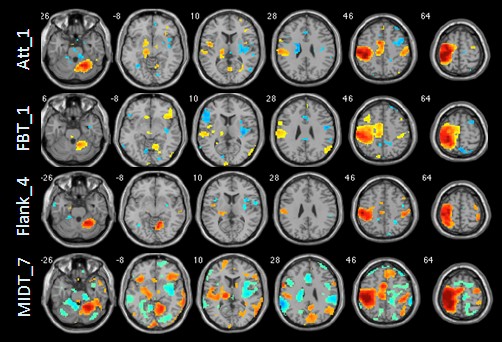


|  | FBT_1 | Flanker_4 | MIDT_7 |
| --- | --- | --- | --- |
| Atten_1 | 0.93 | 0.88 | 0.9 |
| FBT_1 |  | 0.88 | 0.92 |
| Flanker_4 |  |  | 0.91 |

2.


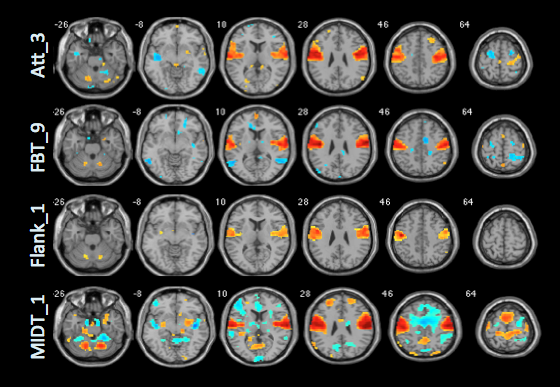


|  | FBT_9 | Flanker_1 | MIDT_1 |
| --- | --- | --- | --- |
| Atten_3 | 0.87 | 0.87 | 0.91 |
| FBT_9 | | 0.93 | 0.93 |
| Flanker_1 | |  | 0.94 |

3.


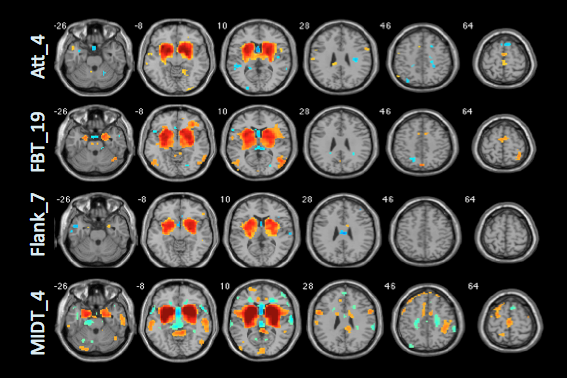


|  | FBT_19 | Flanker_7 | MIDT_4 |
| --- | --- | --- | --- |
| Atten_4 | 0.85 | 0.86 | 0.89 |
| FBT_19 | | 0.83 | 0.89 |
| Flanker_7 | |  | 0.94 |

4.

|  | FBT_4 | Flanker_6 | MIDT_9 |
| --- | --- | --- | --- |
| Atten_10 | 0.82 | 0.85 | 0.93 |
| FBT_4 |  | 0.91 | 0.86 |
| Flanker_6 |  |  | 0.92 |


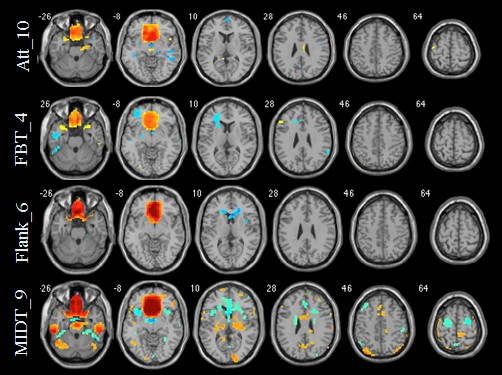


5.

|  | FBT_44 | Flanker_63 | MIDT_43 |
| --- | --- | --- | --- |
| Atten_12 | 0.77 | 0.52 | 0.47 |
| FBT_44 |  | 0.7 | 0.59 |
| Flanker_63 |  |  | 0.76 |


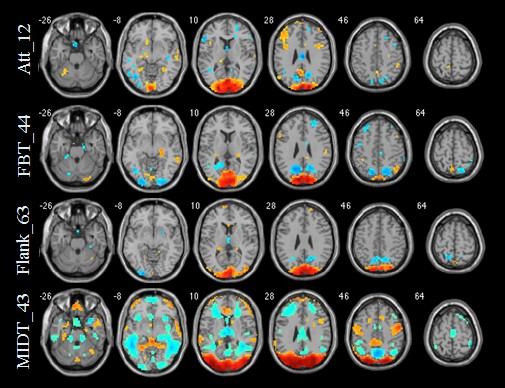


6.

|  | FBT_3 | Flanker_58 | MIDT_44 |
| --- | --- | --- | --- |
| Atten_14 | 0.7 | 0.7 | 0.8 |
| FBT_3 |  | 0.56 | 0.54 |
| MIDT_44 |  |  | 0.65 |


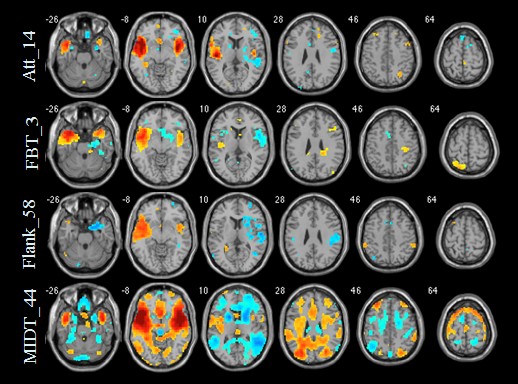


7.

|  | FBT_38 | Flanker_43 | MIDT_50 |
| --- | --- | --- | --- |
| Atten_15 | 0.82 | 0.65 | 0.9 |
| FBT_38 |  | 0.54 | 0.88 |
| Flanker_43 |  |  | 0.62 |


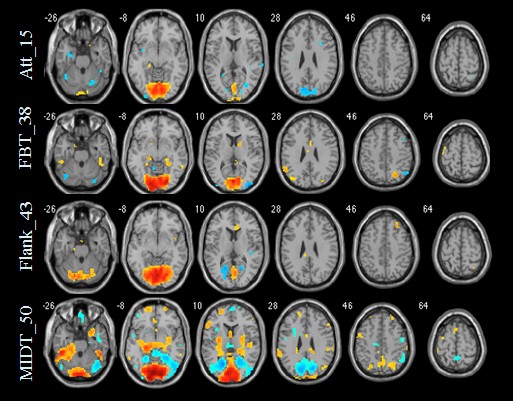


|  | FBT_65 | Flanker_45 | MIDT_22 |
| --- | --- | --- | --- |
| Atten_17 | 0.41 | 0.65 | 0.66 |
| FBT_65 |  | 0.75 | 0.76 |
| Flanker_45 |  |  | 0.84 |

8.


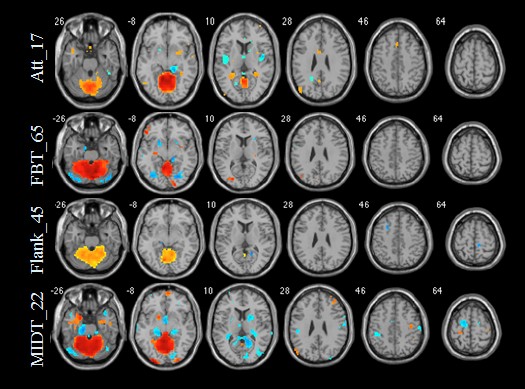


9.

|  | FBT_11 | Flanker_5 | MIDT_37 |
| --- | --- | --- | --- |
| Atten_18 | 0.87 | 0.83 | 0.85 |
| FBT_11 |  | 0.9 | 0.93 |
| Flanker_5 |  |  | 0.93 |


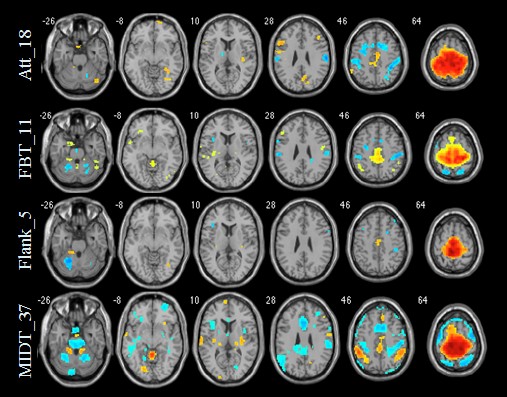


10.

|  | FBT_54 | Flanker_28 | MIDT_51 |
| --- | --- | --- | --- |
| Atten_19 | 0.82 | 0.7 | 0.77 |
| FBT_54 |  | 0.73 | 0.78 |
| Flanker_28 |  |  | 0.68 |


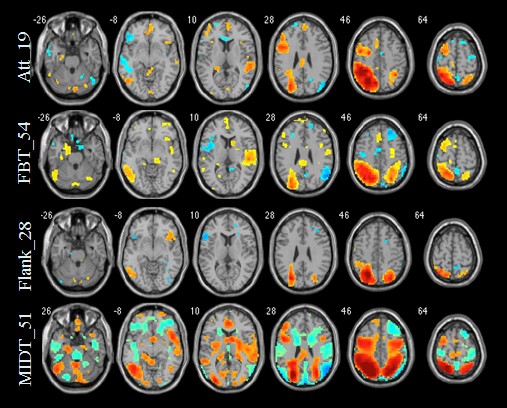


11.

|  | FBT_73 | Flanker_53 | MIDT_67 |
| --- | --- | --- | --- |
| Atten_20 | 0.76 | 0.58 | 0.79 |
| FBT_73 |  | 0.58 | 0.76 |
| Flanker_53 |  |  | 0.63 |


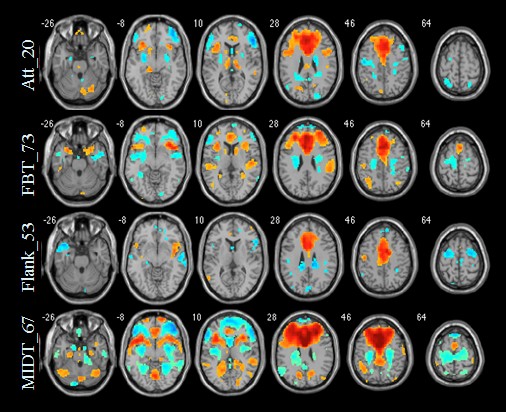


12.

|  | FBT_61 | Flanker_18 | MIDT_20 |
| --- | --- | --- | --- |
| Atten_21 | 0.67 | 0.53 | 0.45 |
| FBT_61 |  | 0.78 | 0.81 |
| Flanker_18 |  |  | 0.83 |


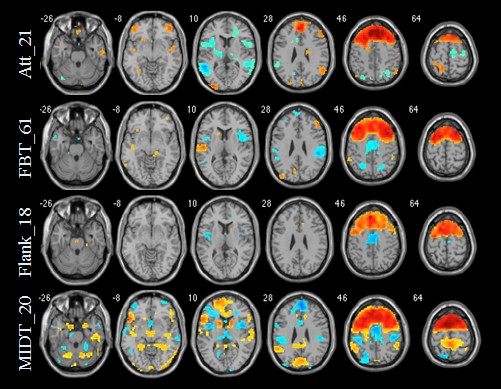


13.

|  | FBT_36 | Flanker_38 | MIDT_62 |
| --- | --- | --- | --- |
| Atten_22 | 0.7 | 0.78 | 0.52 |
| FBT_36 |  | 0.81 | 0.42 |
| Flanker_38 |  |  | 0.56 |


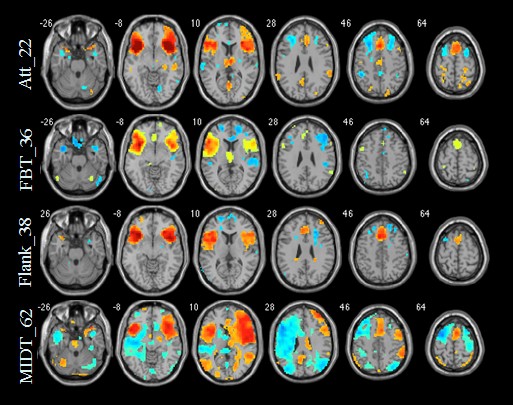


14.

|  | FBT_39 | Flanker_75 | MIDT_14 |
| --- | --- | --- | --- |
| Atten_24 | 0.55 | 0.81 | 0.89 |
| FBT_39 |  | 0.75 | 0.64 |
| Flanker_75 |  |  | 0.87 |


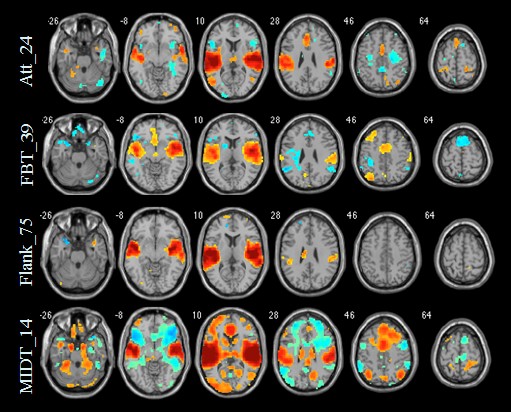


|  | FBT_53 | Flanker_39 | MIDT_61 |
| --- | --- | --- | --- |
| Atten_27 | 0.74 | 0.78 | 0.72 |
| FBT_53 |  | 0.6 | 0.55 |
| Flanker_39 |  |  | 0.7 |

1.
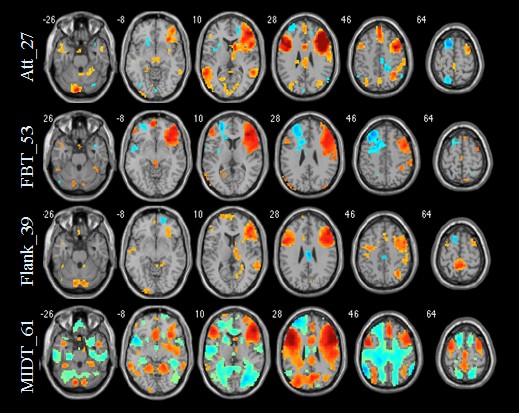


16.

|  | FBT_70 | Flanker_44 | MIDT_63 |
| --- | --- | --- | --- |
| Atten_29 | 0.81 | 0.64 | 0.71 |
| FBT_70 |  | 0.79 | 0.85 |
| Flanker_44 |  |  | 0.86 |


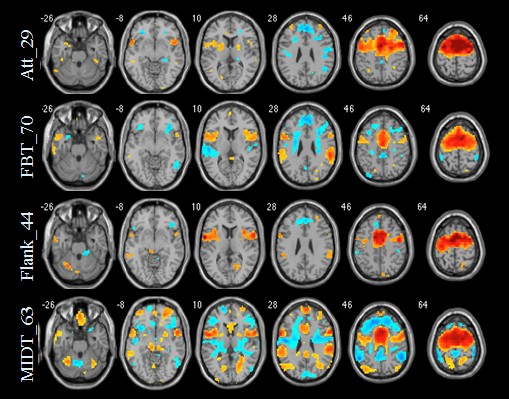


17.

|  | FBT_75 | Flanker_73 | MIDT_35 |
| --- | --- | --- | --- |
| Atten_31 | 0.59 | 0.57 | 0.56 |
| FBT_75 |  | 0.53 | 0.71 |
| Flanker_73 |  |  | 0.83 |


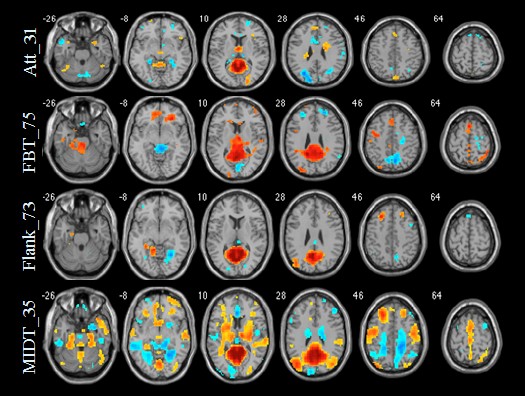


|  | FBT_34 | Flanker_36 | MIDT_18 |
| --- | --- | --- | --- |
| Atten_35 | 0.64 | 0.7 | 0.54 |
| FBT_34 |  | 0.76 | 0.81 |
| Flanker_36 |  |  | 0.65 |

18.


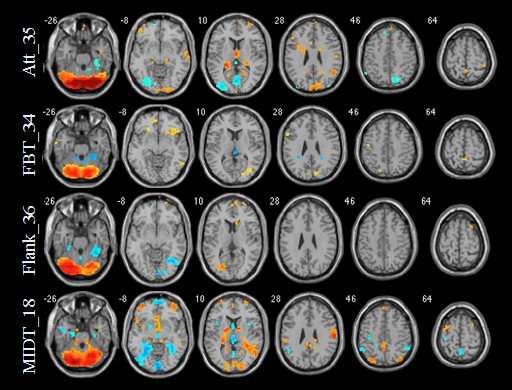


19.

|  | FBT_25 | Flanker_11 | MIDT_47 |
| --- | --- | --- | --- |
| Atten_36 | 0.78 | 0.8 | 0.82 |
| FBT_25 |  | 0.88 | 0.83 |
| Flanker_11 |  |  | 0.83 |


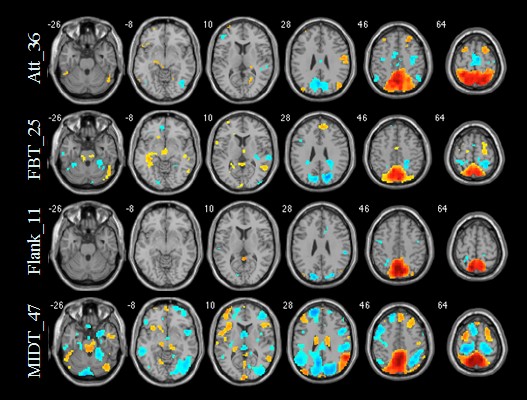


20.

|  | FBT_2 | Flanker_20 | MIDT_8 |
| --- | --- | --- | --- |
| Atten_37 | 0.74 | 0.68 | 0.84 |
| FBT_2 |  | 0.88 | 0.9 |
| Flanker_20 |  |  | 0.86 |


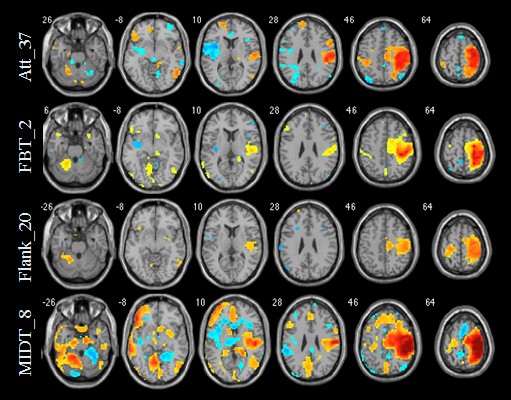


21.

|  | FBT_33 | Flanker_27 | MIDT_52 |
| --- | --- | --- | --- |
| Atten_41 | 0.88 | 0.88 | 0.8 |
| FBT_33 |  | 0.91 | 0.81 |
| Flanker_27 |  |  | 0.81 |


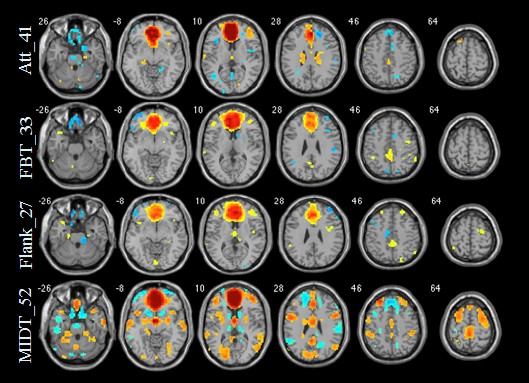


22.

|  | FBT_58 | Flanker_12 | MIDT_72 |
| --- | --- | --- | --- |
| Atten_42 | 0.58 | 0.64 | 0.62 |
| FBT_58 |  | 0.76 | 0.78 |
| Flanker_12 |  |  | 0.86 |

.
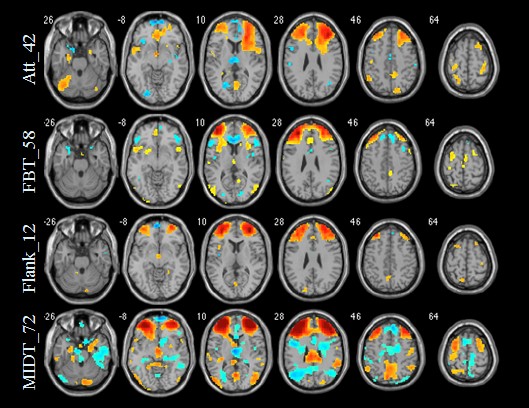


23.

|  | FBT_52 | Flanker71 | MIDT_54 |
| --- | --- | --- | --- |
| Atten_43 | 0.8 | 0.74 | 0.72 |
| FBT_52 |  | 0.74 | 0.66 |
| Flanker71 |  |  | 0.73 |


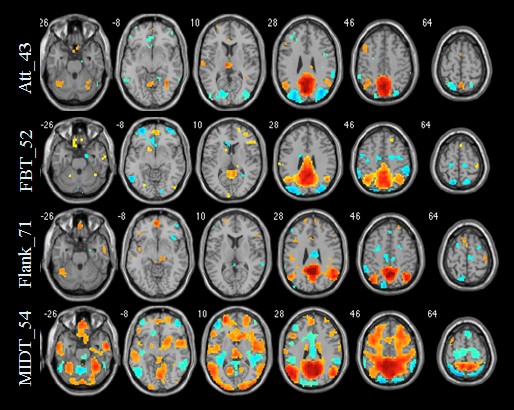


24.

|  | FBT_12 | Flanker_29 | MIDT_38 |
| --- | --- | --- | --- |
| Atten_45 | 0.83 | 0.84 | 0.9 |
| FBT_12 |  | 0.73 | 0.82 |
| Flanker_29 |  |  | 0.94 |


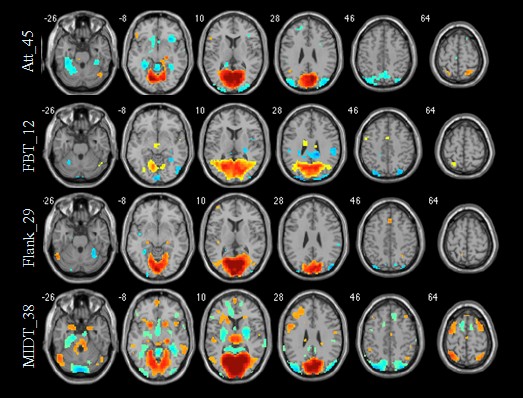


25.

|  | FBT_17 | Flanker_31 | MIDT_65 |
| --- | --- | --- | --- |
| Atten_47 | 0.65 | 0.7 | 0.78 |
| FBT_17 |  | 0.73 | 0.62 |
| Flanker_31 |  |  | 0.68 |


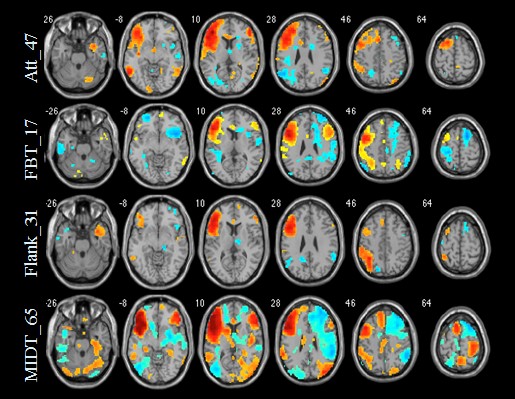


26.

|  | FBT_32 | Flanker_65 | MIDT_31 |
| --- | --- | --- | --- |
| Atten_51 | 0.74 | 0.81 | 0.83 |
| FBT_32 |  | 0.56 | 0.6 |
| Flanker_65 |  |  | 0.82 |


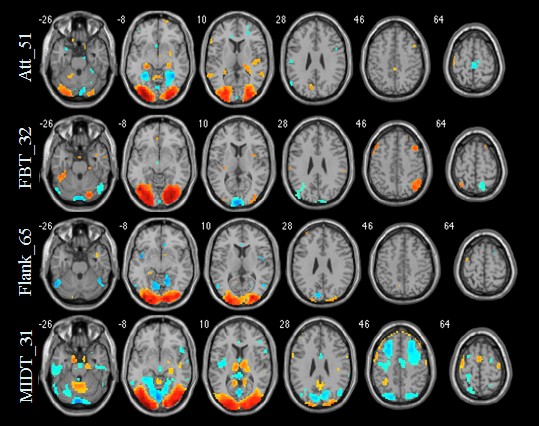


27.

|  | FBT_42 | Flanker_48 | MIDT_70 |
| --- | --- | --- | --- |
| Atten_53 | 0.75 | 0.65 | 0.55 |
| FBT_42 |  | 0.77 | 0.56 |
| Flanker_48 |  |  | 0.56 |


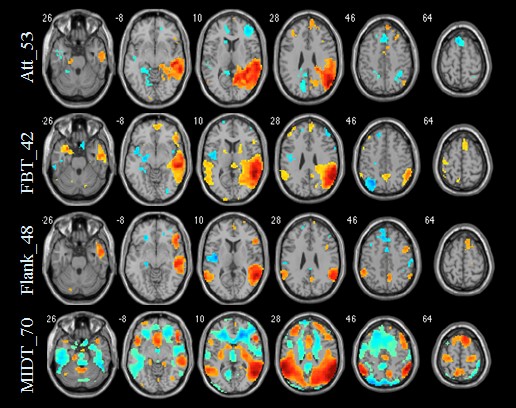


28.

|  | FBT_16 | Flanker_2 | MIDT_42 |
| --- | --- | --- | --- |
| Atten_54 | 0.7 | 0.67 | 0.72 |
| FBT_16 |  | 0.88 | 0.93 |
| Flanker_2 |  |  | 0.95 |


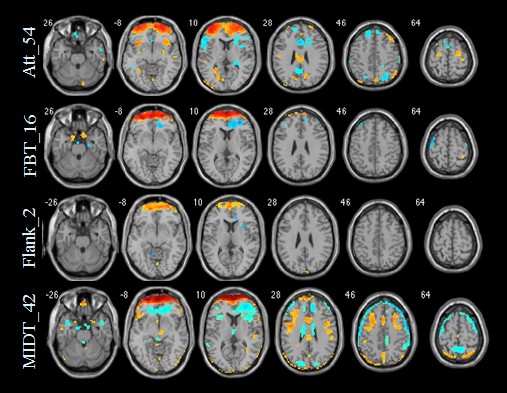


29.

|  | FBT_66 | Flanker_62 | MIDT_25 |
| --- | --- | --- | --- |
| Atten_56 | 0.55 | 0.6 | 0.64 |
| FBT_66 |  | 0.79 | 0.8 |
| Flanker_62 |  |  | 0.83 |


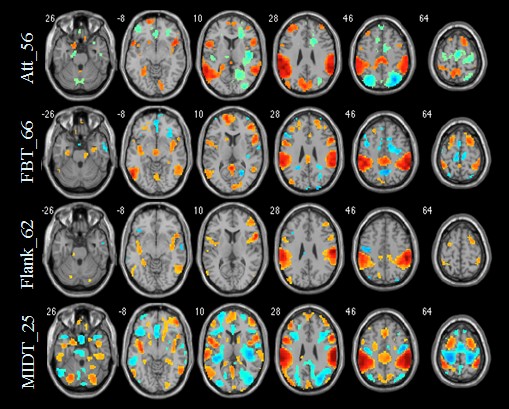


30.

|  | FBT_71 | Flanker_52 | MIDT_60 |
| --- | --- | --- | --- |
| Atten_65 | 0.56 | 0.73 | 0.78 |
| FBT_71 |  | 0.47 | 0.56 |
| Flanker_52 |  |  | 0.67 |


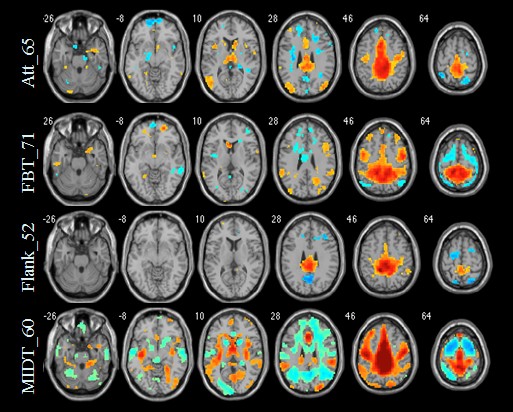


31.

|  | FBT_43 | Flanker_54 | MIDT_40 |
| --- | --- | --- | --- |
| Atten_66 | 0.88 | 0.73 | 0.76 |
| FBT_43 |  | 0.75 | 0.83 |
| Flanker_54 |  |  | 0.85 |


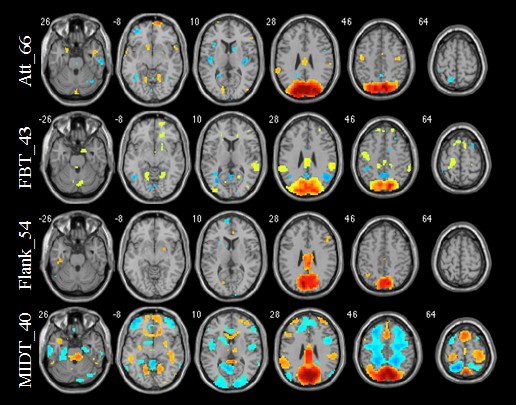


32.

|  | FBT_51 | Flanker_32 | MIDT_39 |
| --- | --- | --- | --- |
| Atten_68 | 0.72 | 0.78 | 0.58 |
| FBT_51 |  | 0.86 | 0.87 |
| Flanker_32 |  |  | 0.83 |


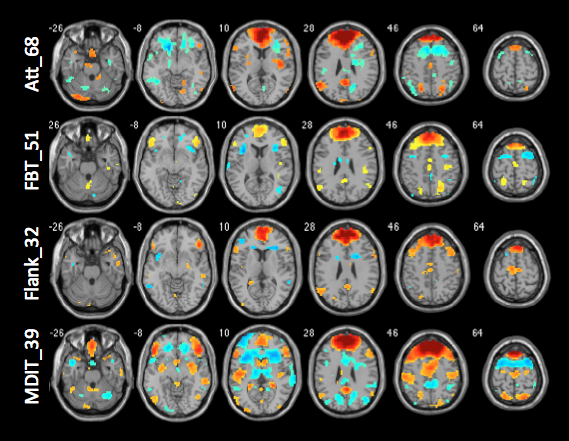


33.

|  | FBT_57 | Flanker_30 | MIDT_66 |
| --- | --- | --- | --- |
| Atten_73 | 0.75 | 0.82 | 0.77 |
| FBT_57 |  | 0.8 | 0.8 |
| Flanker_30 |  |  | 0.83 |


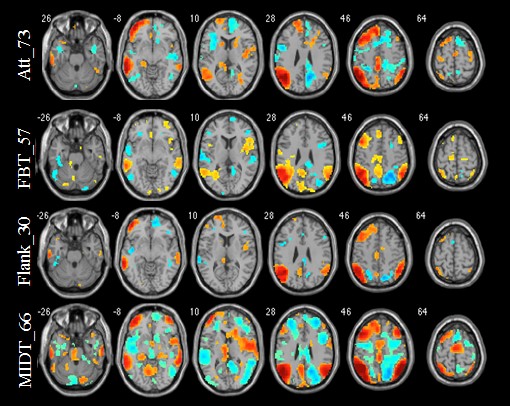


34.

|  | FBT_74 | Flanker_55 | MIDT_56 |
| --- | --- | --- | --- |
| Atten_74 | 0.78 | 0.71 | 0.76 |
| FBT_74 |  | 0.5 | 0.81 |
| Flanker_55 |  |  | 0.5 |


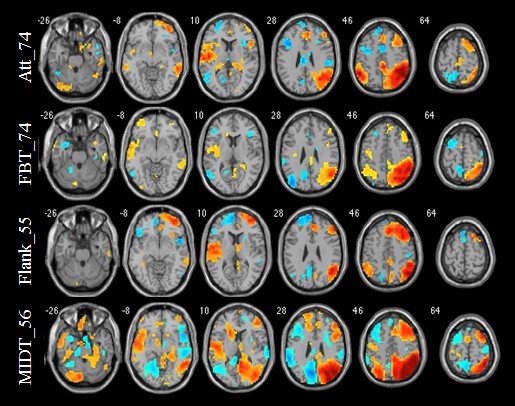


Fig. B. Matched ICs from different datasets. Brain images show matched ICs extracted from the four datasets with minimum correlation coefficient ≥ 0.4. Tables at the right side show correlation coefficients of corresponding matched ICs at the left side. Abbreviation: Att: Attention task; FBT: False belief task; Flank: Flanker task; MIDT: Monetary incentive delay task. Please see figure 9 legend for more explanation.
